# Reflections on a long career in Neuropathology

**DOI:** 10.17879/freeneuropathology-2026-9304

**Published:** 2026-02-23

**Authors:** Michael Farrell

**Affiliations:** 1 Department of Pathology, Beaumont Hospital & The Royal College of Surgeons, Dublin, Ireland

**Keywords:** RCSI, Ireland, Canada, CJD, CTE, Autobiography

## Introduction

It is over 6 years since I was invited to contribute to the Reflections Series in Free Neuropathology. Whilst I was flattered to be invited, I was certain I had very little to say. During my career, I had not kept a scrap book of people or events, nor had I spent too much time polishing my curriculum vitae. But on COVID enforced reflection, I realised that my family might be vaguely interested in what I had got up to. I began by trying to remember and was amazed at how quickly the memories of people, stories and anecdotes came flooding back. I hope that I will not forget or offend too many in what follows below.

## Early years in Ireland’s Northwest

**Figure 1: Map of Ireland’s Northwest.**
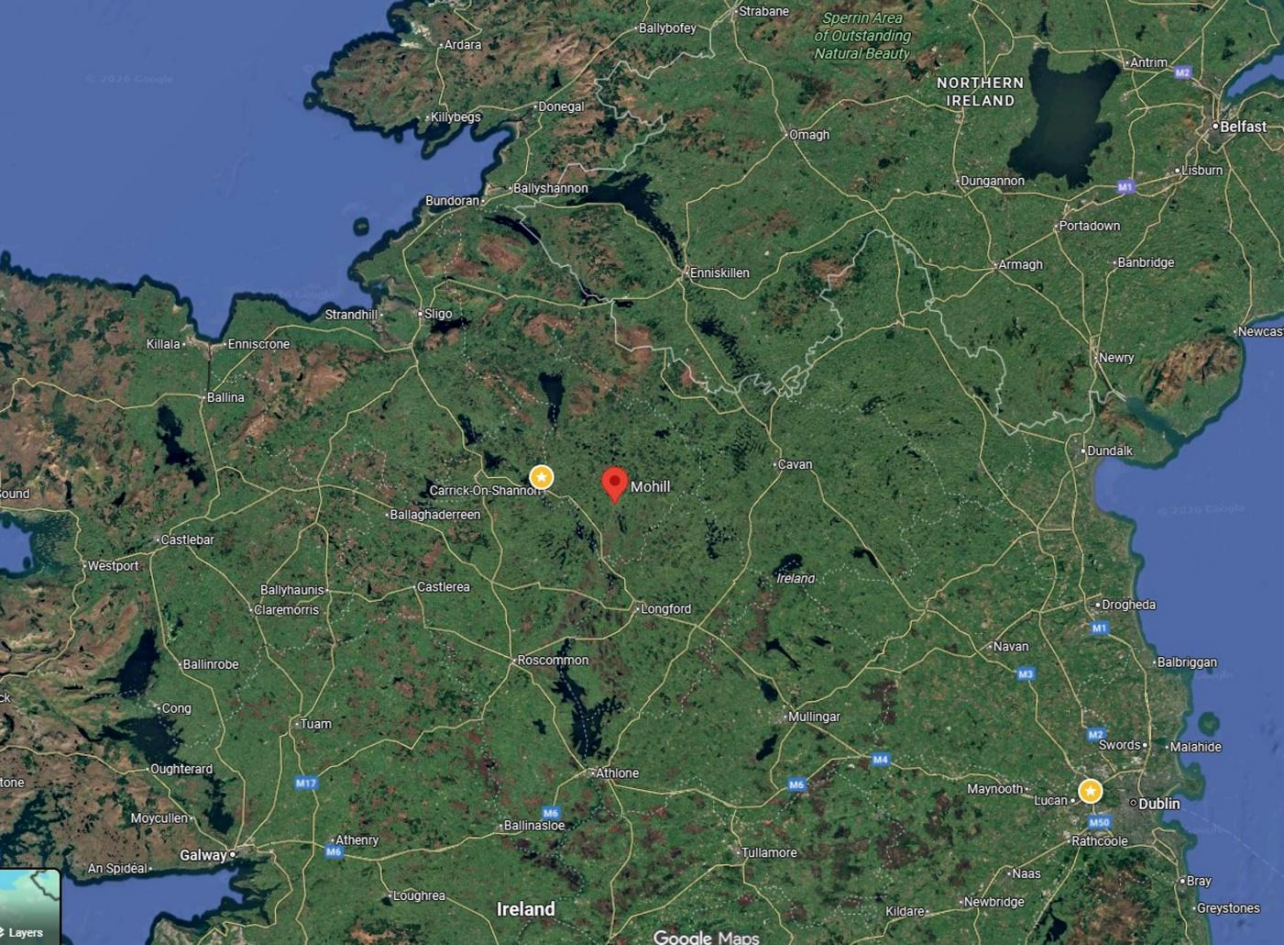


**Figure 2: The author aged 10 with a large haul of Pike caught on Lough Rynn, with his mother in the background.**
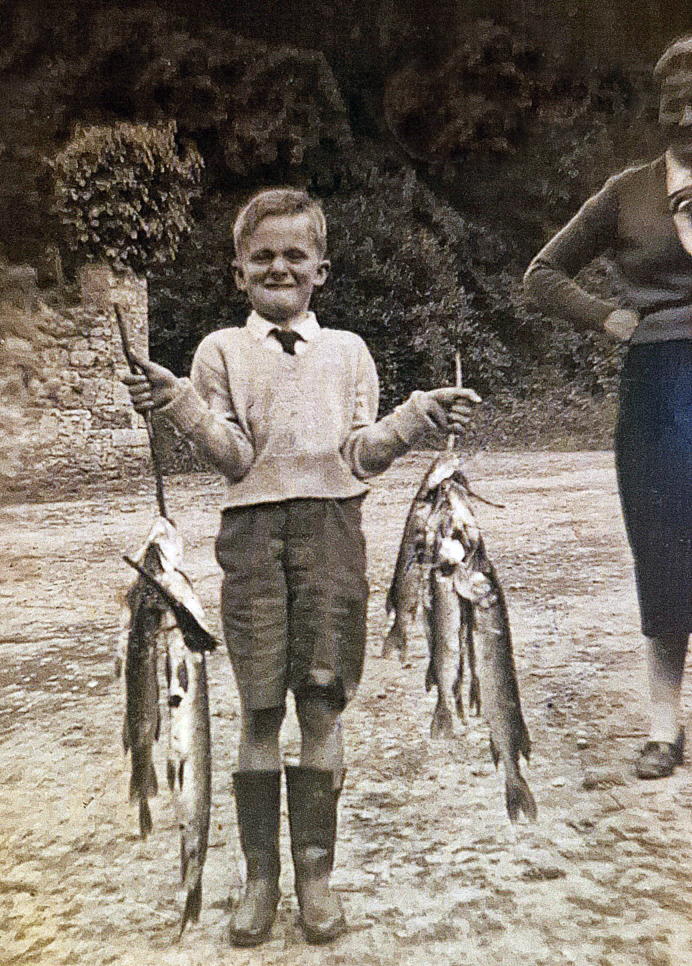


**Figure 3: Surgeon–Lieutenant Dr. Michael J. Farrell June 1944.**
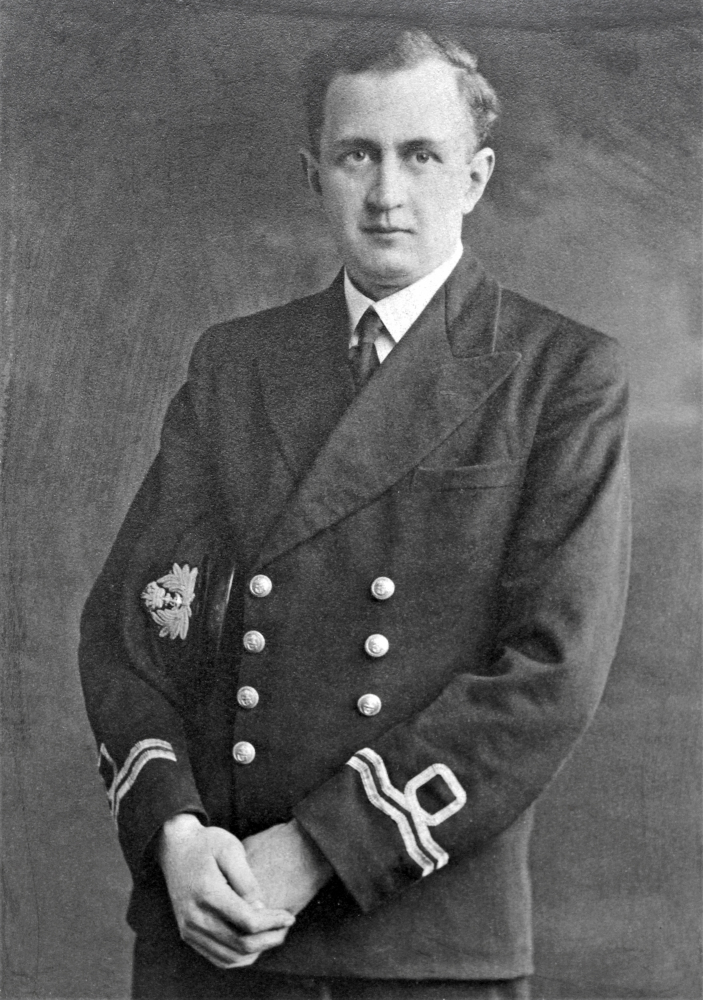


**Figure 4: The Cavan–Leitrim Rail at Mohill in the Late 1940s.**
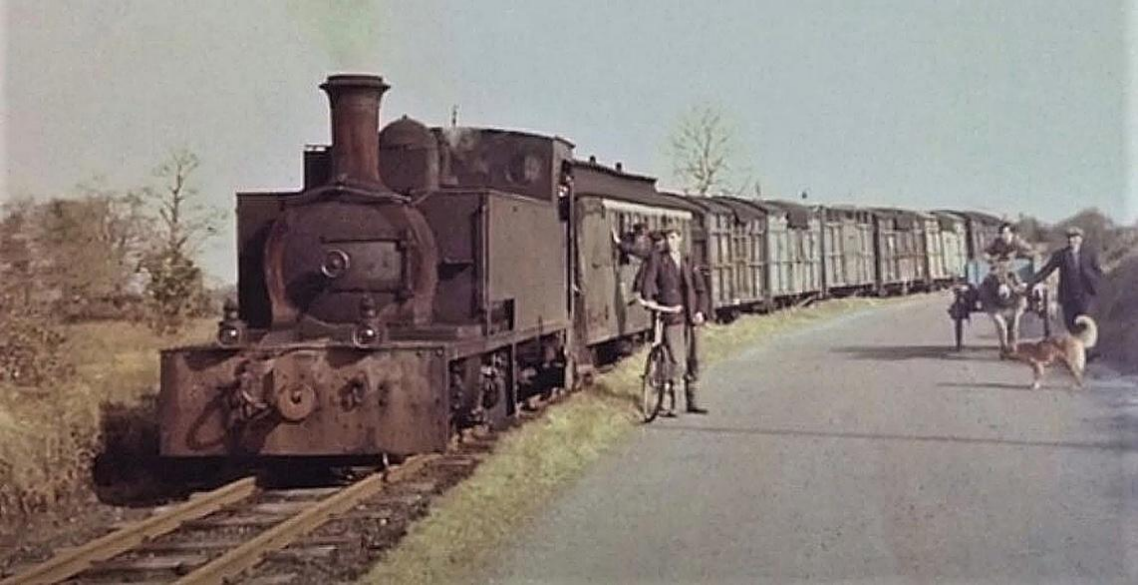


Growing up in a small thriving town in Ireland’s Northwest, **(Fig. 1)** I had little idea of what lay ahead as I spent warm summer days fishing **(Fig. 2)** and trying to play Gaelic football. My parents had settled in Mohill, Co. Leitrim where my father, a Public Health Doctor, was to remain for almost all his working life. During the Second World War he had served in the Royal Navy Volunteer Reserve **(Fig. 3)**. On board the SS Monowai, a converted New Zealand passenger ship, he saw action in June 1944 when the Monowai was deployed to return wounded survivors from the Normandy beaches to the South of England. Later, following surrender of the Japanese in Singapore, he served as Singapore’s Port Medical Officer a job which involved ship inspections and, of course, refreshments in the wardroom of the various incoming ships. Coming to Leitrim in the 1950’s from the affluent southern counties of Carlow and Kilkenny, it must have seemed to my parents they had bought a one-way ticket to Siberia. Leitrim was wracked by emigration. I recall my father, after his daily return from conducting school medical examinations 50 miles away in remote North Leitrim, saying to my mother that yet another school had closed due to emigration. Leitrim’s population fell by a third between the 1950s and mid 1990’s. And yet, the town of Mohill continued as the business centre for a large farming community. Linked to Ireland’s mainline railway system by a steam powered narrow gauge railway guaranteed that Mohill never became isolated, or at least not until a short-sighted political decision was taken to close the local railway in 1958 **(Fig. 4)**. Mohill’s educational system was superb at primary school level, but a girls-only secondary school meant that boys had to choose between the vocational school systems in preparation for an apprenticeship or try to gain entrance to a boarding school where tuition was aimed at the Leaving Certificate with an expectation of a university education thereafter. I had little or no manual dexterity, so an apprenticeship was never a runner. Alternatively, one could take the ferry to England or travel further afield to the USA, never to return. The boarding schools were fee paying and run by religious orders. For some boys they were grim places, in which child abuse was frequent leaving many young men to emerge at 18, scarred for life. I and my 2 sisters were fortunate in that our grandmother’s shares in the Guinness Drinks Empire generated a large enough yield to defray the cost of sending us to secondary boarding school.

## In the footsteps of James Joyce!

**Figure 5: Clongowes Wood College, Co. Kildare.**
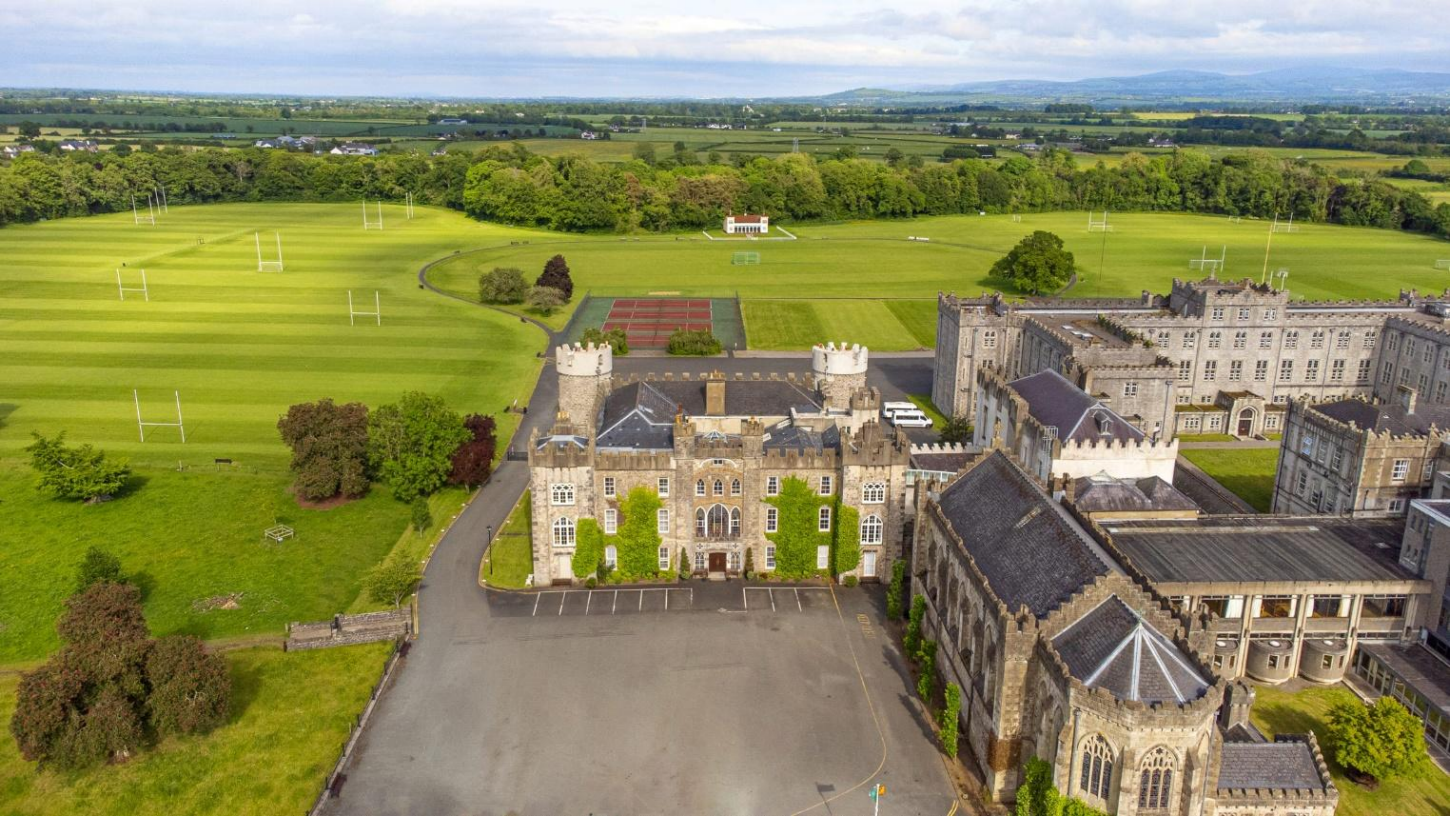


**Figure 6: The 1968 Clongowes First XV. Author standing 4:**
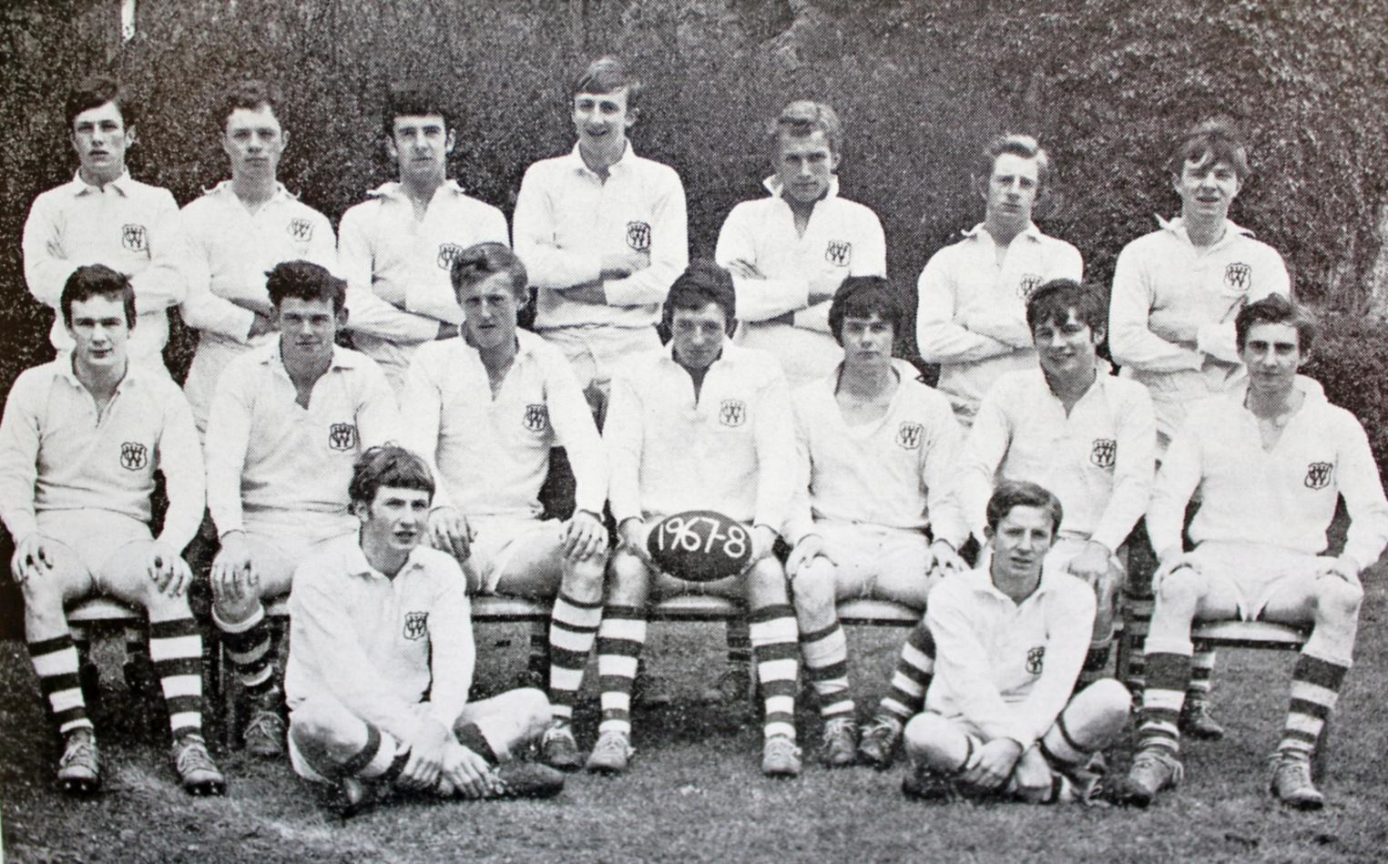


**Figure 7: The authors last day in Clongowes with his lifelong pal, Larry Doyle, helping to pack the authors books.**
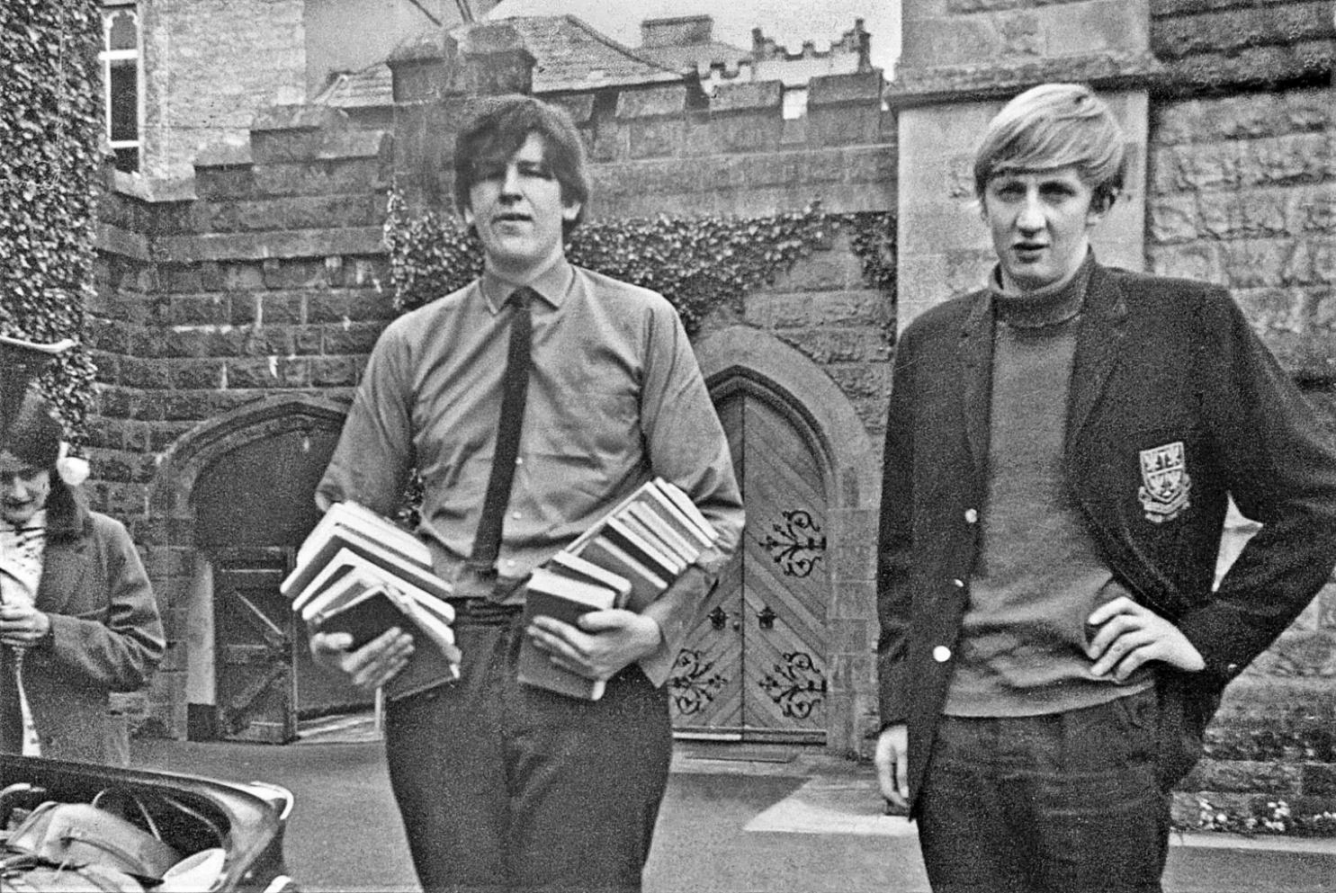


I was particularly lucky in that I was sent to Clongowes Wood College, alma mater of James Joyce **(Fig. 5)** where I spent five happy years, formed friendships that have lasted to today and learned just about enough to gain entrance to medical school. Career guidance in Clongowes was minimal. It was obvious from early days who were the budding engineers, accountants, farmers, veterinary surgeons, horse trainers and doctors. My primary concerns were getting selected for the school’s rugby team **(Fig. 6)** followed by backing horses (one of my classmates went on to train winners of the Epsom Derby and Prix de L’Arc) and making sure I got a fair share of food in the mad scramble at the lunch table. It was at Clongowes that my lifelong interest in rugby football was nurtured **(Fig. 7)**.

## Medical School in the Royal College of Surgeons in Ireland

**Figure 8: The Original Royal College of Surgeons in Ireland Medial School Building as it is today.**
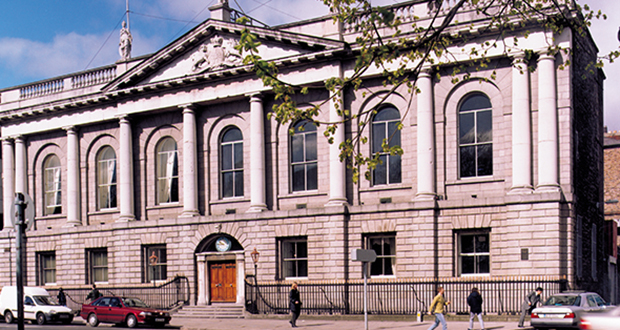


**Figure 9: 118 St. Stephen’s Green, the most recent addition to the RCSI Health Care complex on St. Stephen’s Green, Dublin.**
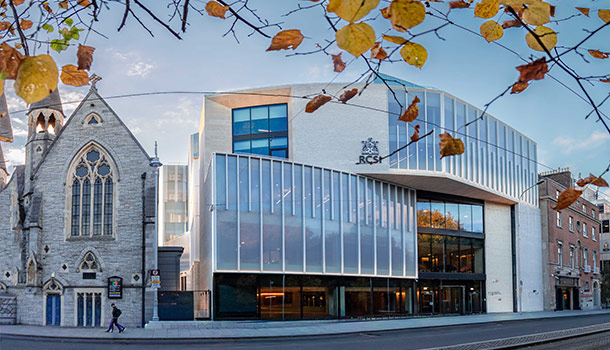


The five years in Clongowes seemed to pass quickly and culminated in my being accepted into The Royal College of Surgeons Medical School on Dublin’s St. Stephen’s Green **(Figs. 8, 9)**. I had an idea of what lay ahead as my father and his brother had both graduated from the same institution, but nothing could have prepared me for the anatomy room smells or the appalling odour of pickled rats and dogfish in Biology class. The pre-clinical years were tough. No sign of a patient, just tedious anatomy lectures and dissections with complex biochemistry formulae thrown in. Were it not for the joys of physiology, I think I would have given up. It was not that the teachers were good or bad, but I just could not warm to learning the anatomical details of the greater and lesser sacs. Physiology was dynamic – there were functions and processes to be learned – it was all so logical. An excellent physiology teacher, Brian Mayne who was also an outstanding physician used to lecture on a Friday afternoon. Without any slides, just an occasional chalked diagram, he effortlessly guided us through the complexities of synaptic activity and axonal conductivity. The fact that he spent almost 3 years in a Japanese Prisoner of War camp added poignancy to his lectures on neuropathy and heart failure. Years later, Brian Mayne’s son Philip and I were contemporaries at London’s Westminster Hospital and later back in Dublin. We often wondered if our dad’s paths had crossed in Singapore as the emaciated prisoners were freed from the appalling Changi prison, where Brian had been incarcerated. At last, with pathology lectures I began to understand illness more clearly and here I came across my first celebrity teacher who was also a neuropathologist.

## Professor Paddy Bofin

**Figure 10: Professor Paddy Bofin, Ireland’s first Neuropathologist and later Dublin City Coroner.**
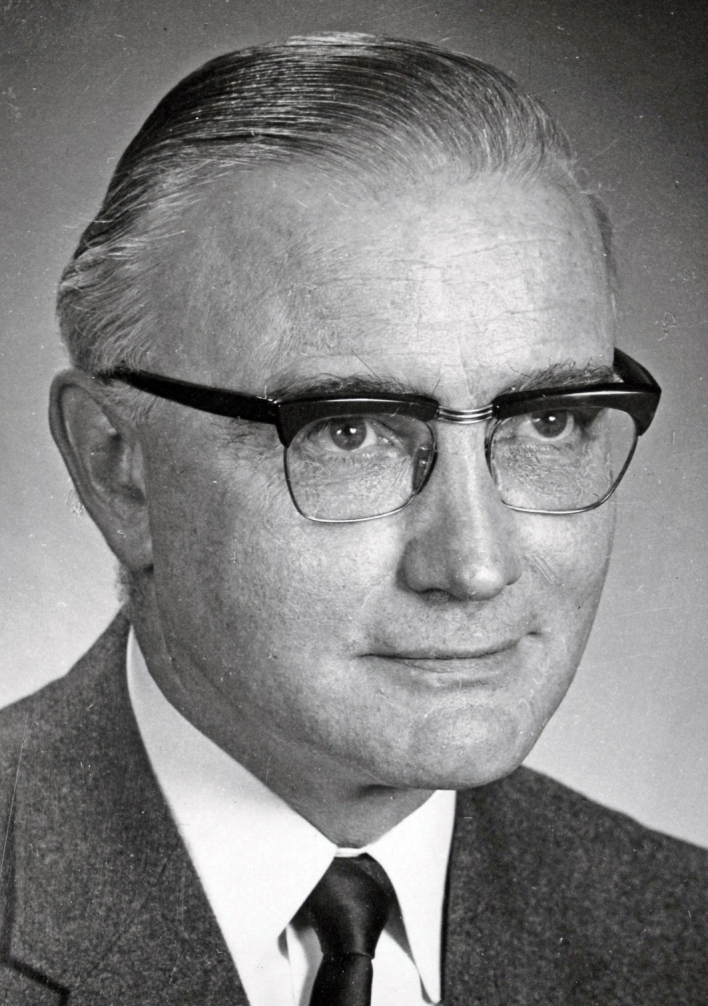


 Professor Paddy Bofin **(Fig. 10)** was a neuropathologist at the Dublin’s old Richmond Hospital; in fact, he was Irelands’ only neuropathologist for much of the 1960’s and early 1970’s. He had a developing interest in Forensic Pathology and later became Professor of Forensic Pathology at RCSI and Coroner to the City of Dublin. Prior to Paddy’s appointment as neuropathologist, autopsies at the Richmond were performed by the neurosurgeons! Paddy smoked a pipe during his riveting pathology and forensic lectures, often using the pipe for theatrical effect. He did not rely on gory slides but instead held the audience in the palm of his hand through a combination of a wonderful Richard Burton-like mellifluous baritone voice and strategic pauses. Many years later, after I had been appointed as neuropathologist to the Richmond Hospital, Paddy, now the Dublin City Coroner decided to teach his successor a lesson. Arriving unexpectedly to the laboratory and a little out of breath, Paddy enquired if he could use his old photography stand. Unfortunately, Paddy had developed a progressive lung disease, which he always attributed to cutting unwashed formalin fixed brains. Anxiously, he told me that two foetuses had been found on Dublin’s Dollymount strand, and furthermore that the police knew the unfortunate woman who had suffered the miscarriage. *“Would you mind terribly taking a few photographs for me”* Paddy enquired. Off we went to the postmortem room where I carefully positioned the two foetuses and the single placenta beneath the arc lights on the dissection table. I made sure that the pictures, taken on Ektachorme 35 mm film would be perfect for Paddy. As he departed with the roll of film, he conspiratorially whispered that he might be dealing with another “Kerry Babies situation” **[1]**. I forgot about the case but 6 months later a colleague who had attended one of Paddy’s presentations at an International Forensic Pathology Meeting in Belfast told me that Paddy, while illustrating his talk using my slides explained that *“an eminent neuropathologist had failed to recognise the foetuses as canine”*. I was mortified. Paddy enjoyed telling the story for years afterwards and from that moment I learned to open my eyes, to see and not just look! Paddy was a brilliant storyteller and for many years was contributor to Sunday Miscellany, a popular Sunday morning Irish Radio Programme in which contributors would read their essays on all aspects of life. Paddy’s contributions, of which there were many, including meeting Muammar Gaddaffi, the Dublin Bombings and Christmas on the wards of the Richmond Hospital, were recently discovered by his son Conor, who has brought Paddy’s stories back to life as part of a podcast series, called ‘Tales of a Dublin Doctor’ where they are read by Conor. 

## Summer of ‘72 in Minneapolis and the patchwork mouse

**Figure 11: Professor Robert A. Good. Professor of Paediatrics, Immunology and Pathology, University of Minnesota.**
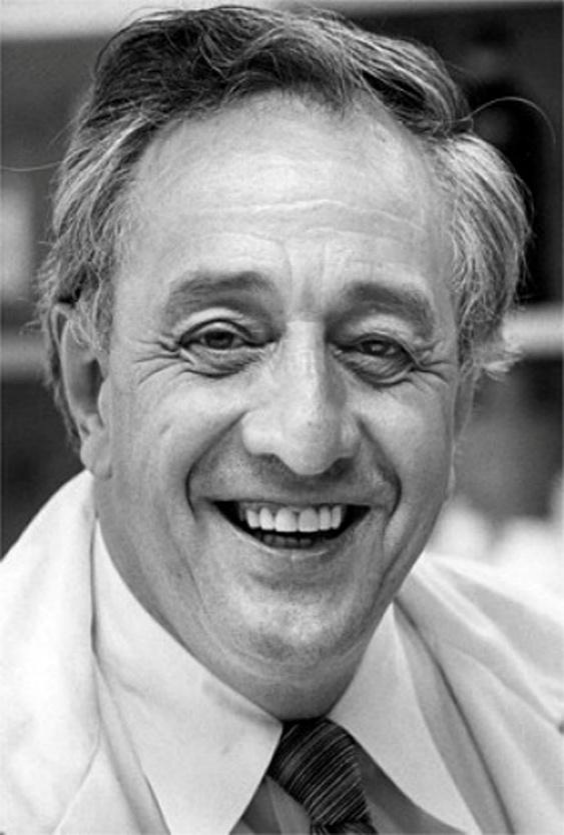


 Pathology teaching in RCSI was and remains outstanding and I am enormously proud to have been part of the RCSI pathology department for over 40 years. Back in 1972, having passed the pathology examinations with distinction I was awarded a summer scholarship to the University of Minnesota to work on a project in the laboratory of Robert Good **(Fig. 11)**. Funding for the scholarship was raised by an RCSI Graduate, Dr. Stacey B. Day who looked after me over those summer months in Minneapolis. The project involved an examination of the systemic changes induced in the immune system by a small, localised burn. The project might not pass through an ethics committee today, but nevertheless important findings were generated and led to my first publication **[2]**, with Bob Good as co-author and which I am certain opened many doors for me in later years. The University of Minnesota Pathology department was a hive of immunology research led by Bob. To most, he was the founder of Immunology, conducting ground-breaking studies that helped show the pivotal role played by thymus in immunity. In 1968, he performed the first successful bone marrow transplant. Summer of 1972 was an extraordinary time in America. On my second night in Minneapolis, I watched as the evening news described in low key terms, there had been a break-in at the Democratic Party headquarters at Washington’s Watergate Hotel! 

**Figure 12: Robert Good on the cover of Time Magazine March 19:**
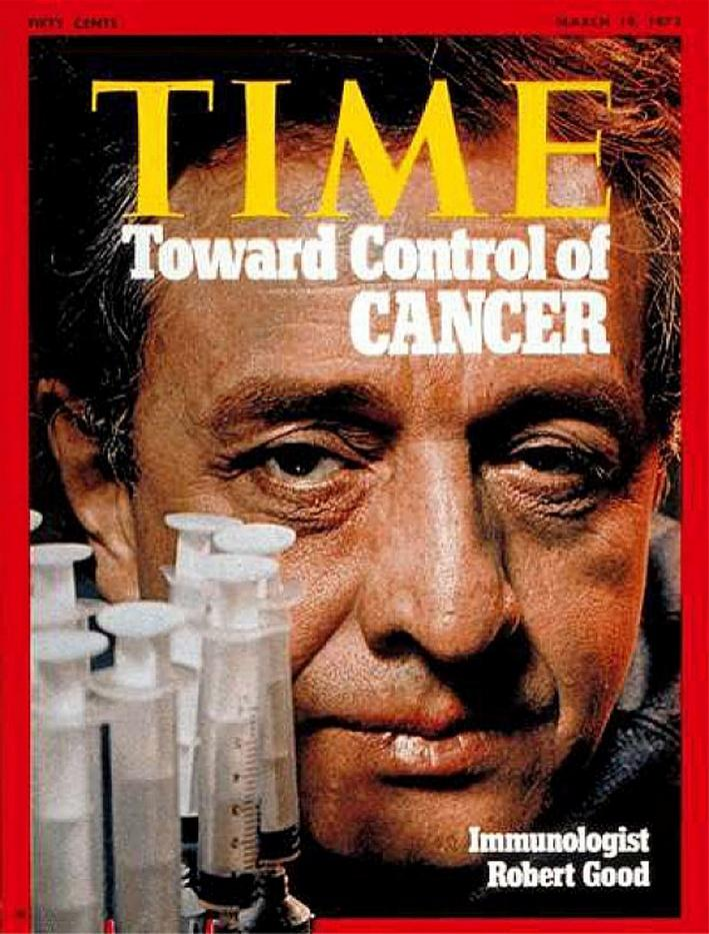


**Figure 13: The Patchwork Mouse by Joseph Hixson, Published 1976.**
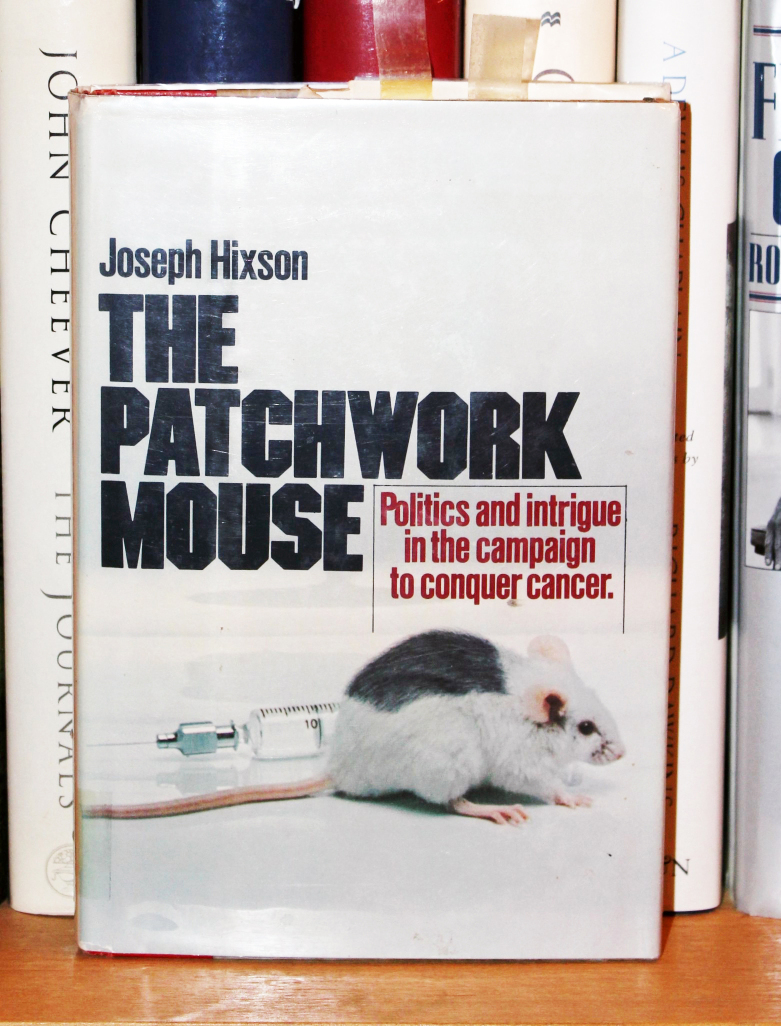


Towards the end of my elective, there were rumblings in the Department of Pathology that Good and team were about to move *en-bloc* to head up New York’s Memorial Sloan Kettering Institute and where he would eventually become president and director. I was anxious that I completed my project before Bob moved. In a next-door lab was Bill Summerlin, a tall Texan dermatologist, who was one of the most stimulating physicians I ever met. He regularly showed me his amazing mice on whom he had transplanted skin grafts from genetically unrelated donor mice without immune suppression. *“Hey Irishman – let me show you something”* he would call out as he showed me his patchwork mice. Eventually the entire immunology team including Bill moved to New York as predicted. My mother who was always proud of her children’s achievements, showed me the March 1973 Time magazine featuring Bob Good on the cover **(Fig. 12)**. *“Isn’t that the man you worked with last summer?”* I hadn’t the heart to tell her that the medical research world had come crashing down around Bob and Bill. It was a sad time for all and is brilliantly described by Joseph Hixson in his famous book “The Patchwork Mouse”, published in 1976 **(Fig. 13)**.

## Graduation, internship and learning to see

**Figure 14: The Richmond Hospital, Dublin.**
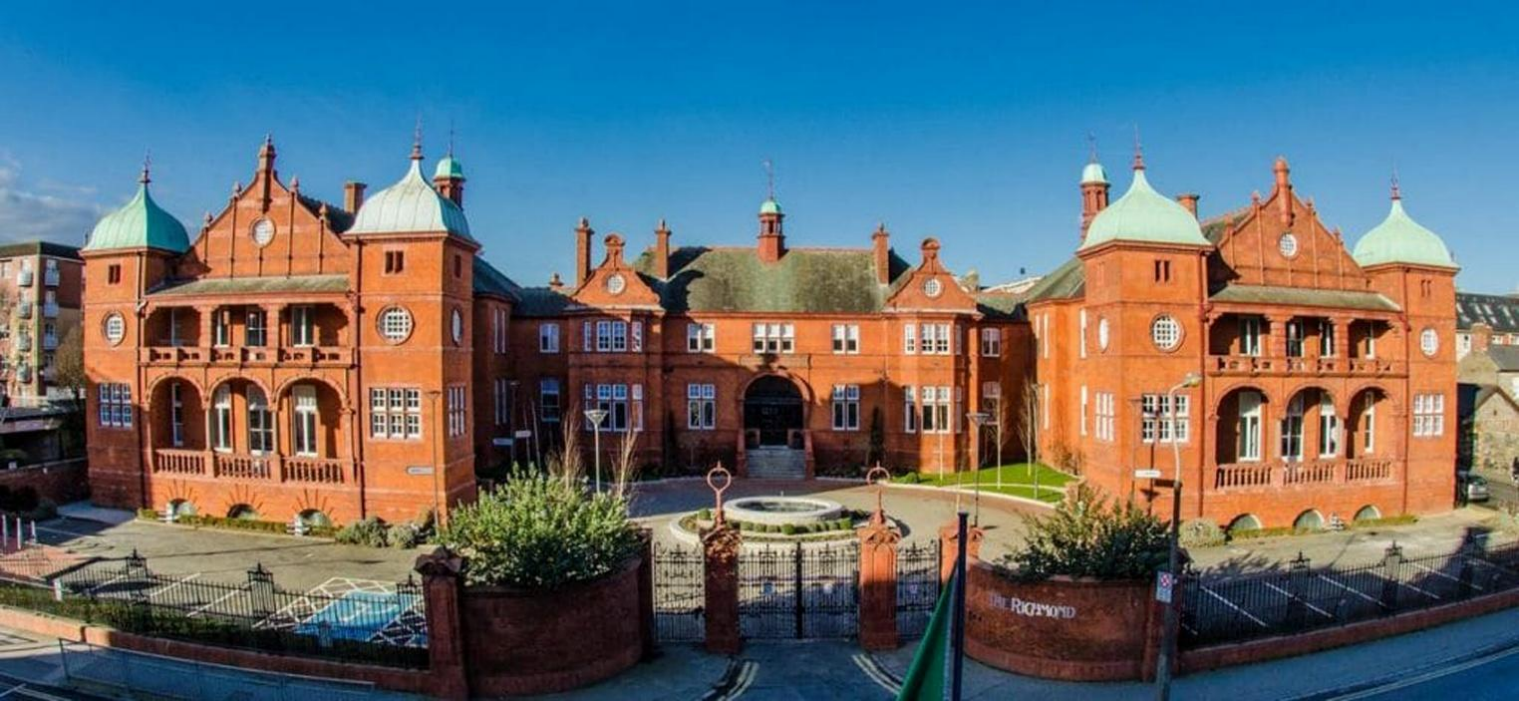


**Figure 15: Dr. Harry Counihan, Chest Physician, Richmond Hospital 1987.**
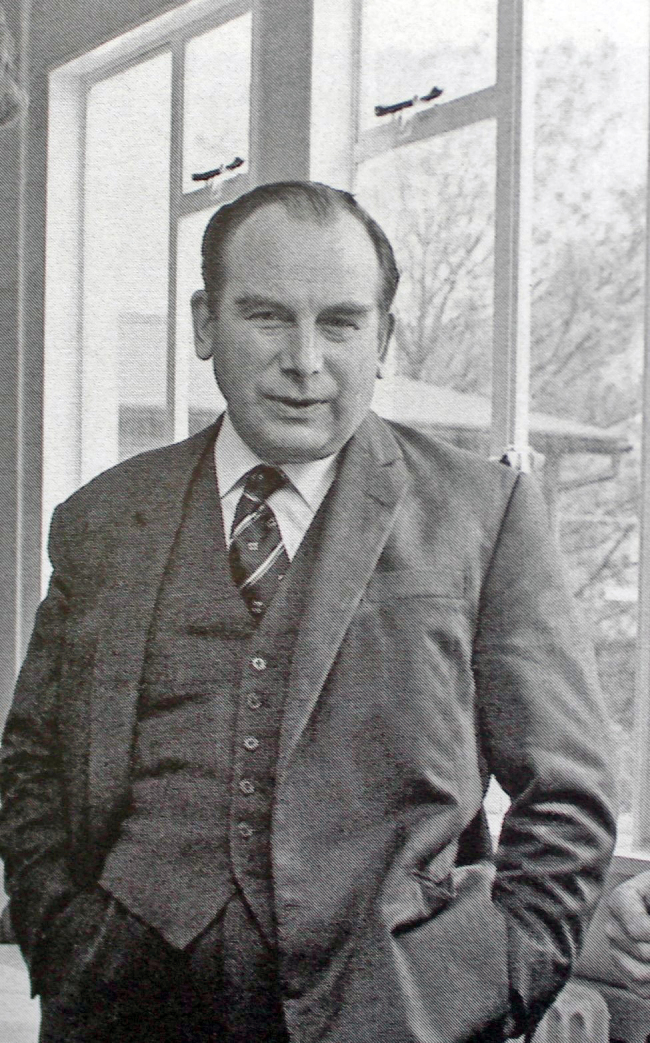


The final two years in medical school were dominated by clinical attachments and bedside teaching across all the major specialties. Dublin’s great history of clinical medicine as exemplified by Stokes, Graves, Adams, Corrigan and others was continued by the surgeons and physicians who bestrode the wards of the Richmond **(Fig. 14)** and Jervis St Hospitals, just as their forebears had done over a century previously. Small bedside groups of not more than twelve students were asked by the consultant to take a history or conduct a particular examination. Patients were extraordinarily compliant and freely gave of their stories to help educate the students. I don’t know if we ever appreciated how much we learned from the patients who were always, no matter how sick, willing to talk about themselves. Medical student clerking required that we interviewed and examined medical and surgical patients and present our findings at the bedside to the consultant. It didn’t always go smoothly. Dr. Harry Counihan **(Fig. 15)** who was the best medical teacher I ever had, rarely spoke. As a student, and later when I spent 6 months as his medical intern, I learned to be concise and to the point and never to expect praise. The fear of not meeting his expectations was enough to make me strive to be as thorough as possible. It was a trait that later was to serve me well in pathology. However, there were occasions when I did not match his expectations, most notably when I had clerked a new patient who had some form of industrial lung disease. I had taken a history, noted the patient’s finger clubbing, organised a chest x-ray and arranged preliminary pulmonary function studies but did not produce a cause. Dr. Counihan, my future mentor simply asked the patient where he worked. I had already figured out that the patient worked in a factory that made powder-based cleansing agents but had thought no more. *“Farrell, call the Poison’s Centre in Jervis St and see what’s in that stuff”*. I did as I was told and minutes later, sheepishly returned to the bedside ward round, having discovered through a simple phone call, that the *“stuff”* was full of crystalline silica! Again, I learned another valuable lesson, to leave no stone unturned. Harry Counihan remains legendary in Irish medicine and in a fitting appreciation, his colleague and friend Harold J. Brown said on the occasion of Harry’s death that he was *“one of the most respected, trustworthy and ethical physicians of his generation”*. I was fortunate to have Harry as a life-long mentor.

## Obstetrics & Gynaecology – Not for me

**Figure 16: Dr. Tom Farrell, GP Middlesborough, England following a domiciliary delivery.**
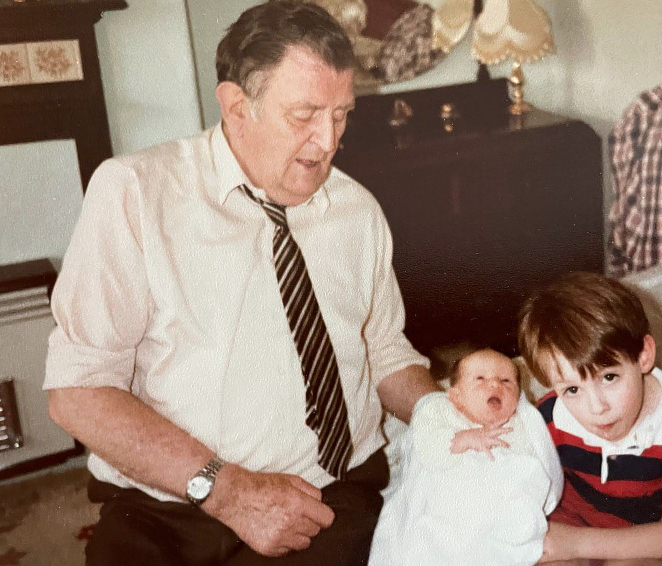


As we glided through the clinical rotations, I began to think of the specialties I would avoid in the years ahead. Obstetrics – Gynaecology, especially the former, was brilliantly taught in Dublin’s Coombe Lying in Hospital which together with Dublin’s Rotunda and National Maternity Hospital, continue to serve the women of Ireland for over 250 years. Even though the Master of The Coombe allowed me to perform several deliveries including showing me how to apply a pair of Kielland’s forceps, the specialty never interested me. My uncle, the late Dr. Tom Farrell **(Fig. 16)** also a former student at the Coombe Hospital and himself responsible for thousands of deliveries in the North-East of England, tried to influence my decision by presenting me with his personal Kielland’s forceps! Likewise, psychiatry never appealed as it took a very long time to bring about an improvement in a patient’s life. As an intern, I and the Senior Registrar had to tell a young woman who was in the respiratory ward with asthma, that her husband had killed himself and two of their children that morning. Her face, as we conveyed tragic news will remain with me until I die. I never had the temperament or manual dexterity for surgery and so as I worked my way through intern year, doing 6 months medicine and 6 months surgery, thoughts moved to post-graduate training.

**Figure 17: Professor Seamus Dundon, Professor of Paediatrics, Royal College of Surgeons in Ireland, 1975.**
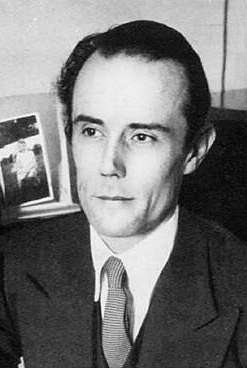


I had already decided to try to obtain a good foundation in medicine and so began 6 months of paediatric medicine. Now that was tough, including as it did, time in the oncology ward where delivering chemotherapy to children, often by intrathecal injection was terrifying. It would have been easier if we had already established a relationship with the children, but it just wasn’t possible to have the medical oncology trainees’ on-call round the clock, and so the rest of us had to fill-in. The children, never having previously seen us, always associated our arrival with pain. Having somehow managed to pull off first place and a Gold Medal in Paediatrics I was allocated to work with Seamus Dundon **(Fig. 17)**, Professor of Paediatrics. He was a gentleman in every sense. His forte was to distract the sick child and observe. He exemplified masterly inactivity with cat-like observation or MICLO as it is usually termed. Unable to get the same co-operation from the sick children, I eventually just asked *“’How do you do it?’ Michael”, *he replied*, “I never stare at child’s eyes”.* I never again had a problem getting a child’s co-operation. During the 6 months in paediatrics, I regularly ran into a paediatric neurologist, Dr. Niall V O’Donohoe who was on the verge of an outstanding international career in epilepsy. A stickler for detail, he provided my first consistent exposure to clinical neurology. As the 6 months rotation drew to a close, he suggested I might consider a career in paediatrics and come work with him. I was chuffed and thought long and hard about his invitation but reluctantly decided to continue on the path of getting a good foundation in general medicine. Years later, when we became neuroscience colleagues and friends, I told Niall, how much his invitation meant to me.

## From bedside to bench

**Figure 18: Headline:**
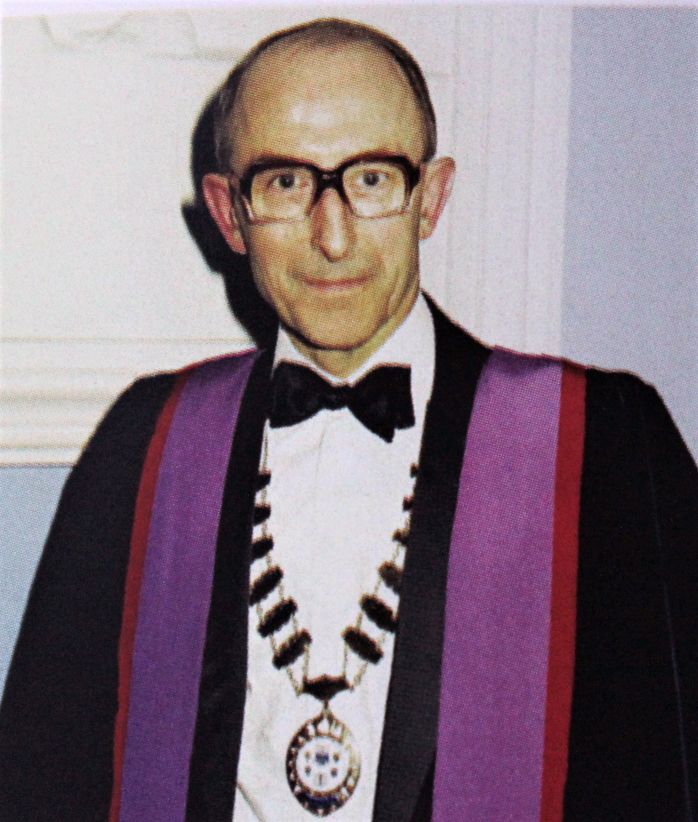


And so back to medicine, where I was to spend another 18 months, all the time trying to make my mind about a career. More in desperation than love, I decided to try surgical pathology, at least for a few months, just to have time to think as I was a little burned out after studying for the MRCPI post graduate examination in medicine. It was on the advice of Dr. Bob Towers **(Fig. 18)**, a brilliant old school pathologist that I stayed on to finish medical training. *“Come back next year after you have passed the medicine exam – it will stand to you in the long run”* – how right he was! My career in pathology began on the morning of July 1^st^, 1976, having just come off 24 hours medical emergency call! The transition from a busy clinical service to a desk job was abrupt. The first weeks in pathology were bewildering as I adapted to the lower pace and struggled to remember any normal histology. The department was busy but by today’s standards was inadequately staffed at every level. One could not have asked for better teachers than Bob Towers, Mary McCabe and John Dinn, all now sadly deceased. Immunohistochemistry was yet to become widely available. All diagnoses were based on haematoxylin and eosin-stained sections. I struggled, but with outstanding teaching from Bob and Mary and an experienced senior registrar in Joe Stuart I began to find my feet. For my first autopsy, I was advised to make my way to the postmortem room – which was found at the end of a long dark underground tunnel – and where the autopsy staff would look after me. If I had not reappeared by 5pm Bob or Mary would come looking for me! I learned so much from the autopsy technicians which helped me in the years to come. Forty-eight years later I still visit that same mortuary every Wednesday morning for brain examinations. The current autopsy technicians are as kind and as expert as were their predecessors when I began my career. Little by little my confidence began to grow. As the hospital had a neurosurgery department and a neuropathologist, I began to take in interest in the neuro specimens, but my primary interest was in passing the MRCPath pathology examination which was typically taken at the end of 5 years.

## Off to London England

**Figure 19: Professor Kristin Henry, Professor of Pathology Westminster Medical School, London.**
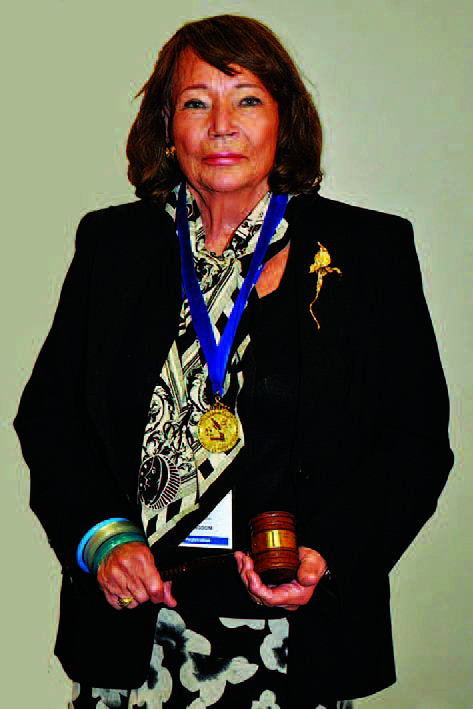


Working in the UK was at that time essential for success in the MRCPath examination and so my wife and I packed our small car and headed for London, England. Our time there was tough involving a long commute to work, birth of our first child and preparation for the dreaded examination. But the work was busy and rewarding with many interesting cases, good teaching especially from DH Mackenzie, Kristin Henry **(Fig. 19)** Isobel Filipe and Tony Branfoot, as well as friendly and brilliant colleagues such as the late Jeremy Jass. The stress of breast frozen sections was and is still the stuff of nightmares. No pre-operative fine needle aspirations – no pre-operative mammography – just a brilliant breast surgeon called Harold Ellis emerging from the operating room clutching a breast lump and watching as I cut and stained the frozen section. Together, we looked down the double header microscope before Harold, based on my opinion, would return to close up or to conduct a mastectomy. Unfortunately, my Dublin boss had written a glowing reference which generated the expectation that I was very experienced. My new London boss, Professor Mackenzie decided I was competent enough to interpret the frozen sections without too much supervision. The stress of 4 weeks continuous breast frozen section service during that hot 1979 July in central London was too much, and I began to develop headaches, which were the start of lifelong migraine. Years later, a neurology colleague whose opinion I sought, replied *“Listen Michael, I am classy neurologist, I don’t see people with headache, but like you I also have migraine which generally only affects intelligent people like me and you!”* For exposure to Neuropathology, I visited Maida Vale Neurosurgical Hospital where Robin Barnard kindly taught me more than enough neuropathology to handle any MRCPath questions about the brain.

## The dreaded MRCPath examination and aftermath

There was little time to enjoy London life as all efforts were aimed at passing the MRCPath examination. Taken in a busy South Coast Hospital, the exam was tough. Another candidate and I fearing we had done badly on the first day’s autopsy and surgical cases went on the beer. The following day’s specials and viva are vaguely remembered. The external examiner, Roger Cotton, whose book on surgical pathology I had learned word for word was tough but fair. Maybe our “relaxed” state resulted in the unbelievable news that I had passed, as did my beer drinking fellow examinee. Celebrations included a rare pizza night out with my wife and daughter in Hampstead, after which, while running down Hampstead Hill, I hit the kerb and launched daughter’s buggy and myself into mid-air, both of us landing upside down on the pavement, unhurt. At this time, my love of clinical medicine began to re-emerge.

## Canada beckons

**Figure 20: Dr. Sean Murphy, Neurologist, Richmond Hospital and Beaumont Hospital, Dublin.**
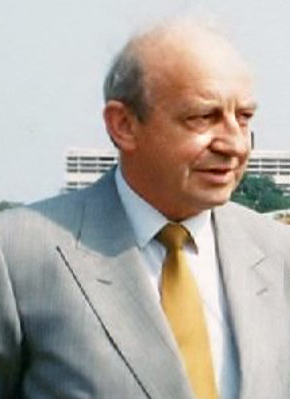


I now had time to think and consider my career options. I didn’t feel ready to look for a consultant Histopathologist post. I wasn’t sure what subspecialty, other than neuropathology interested me. UK training positions in neuropathology were geared towards the specialist examination in neuropathology. Having a surgical pathologist who had already completed the general surgical pathology examination, assume a neuropathology training position was rare. Anyway, I was restless and wanted to escape London life. Word came through that my old Dublin teacher, Paddy Bofin was unwell and that there might be an opening for a neuropathologist in my former undergraduate teaching hospital, in a few years’ time. Sitting in our small North London maisonette a call came through from Sean Murphy **(Fig. 20)** in Dublin. Sean was an outstanding US trained neurologist who had worked with Denny Brown, Dan Drachman and Norman Geschwind in Boston. Everyone knew Sean, who along with Hugh Staunton, Eddie Martin and Michael Hutchinson had led the way in Dublin and Irish Neurology since the early 1960s. *“Farrell, I hear you are thinking of doing Neuropathology – I have a pal in London, Ontario who might be able to fix you up with a residency in Neuropathology”*. I did not know what to say, as already I had a preliminary chat with the University of Toronto Pathology Department, about doing a Neuropathology Fellowship there. Starting a residency again was going to add another 3- or 4-years training on top of time already spent in general medicine and in Histopathology. My wife Sandra was supportive of a move to Canada either way but would probably have preferred the glamour of Toronto. However, working as a resident rather than a fellow, I felt that I would get more learning experience, a suspicion that was confirmed in years to come as I watched visiting fellows and residents compete to scrub alongside Charles Drake, the world’s greatest vascular neurosurgeon at London Ontario’s University Hospital.

We made our minds up to go to London in Southern Ontario. A few days later after welcoming phone calls from John Kaufmann and Henry Barnett, we put our apartment on the market and made travel arrangements. Our last Christmas in London, UK took place in the middle of “The Big Snow” of 1982 with a temperature of –15 ℃ recorded on December 15^th^. We re-packed the same small car that had brought us to the UK from Ireland, this time with our daughter and her toys and headed up the snow-covered motorway hoping to make the Dublin bound Liverpool ferry. It was a nightmare trip. Blizzard conditions meant that roads were blocked, but fortunately, I had a cousin who lived along the route and who kindly provided us with accommodation until the snowstorm had abated. Eventually, we reached Liverpool where there was a 3-hour wait before we could board the ferry. How the three of us didn’t die from hypothermia, I will never know. Arriving in Dublin it was as if we had landed on the moon. Frozen snow lay everywhere – roads had not been cleared. Mobile phone communications didn’t exist – there was no help in the event of a breakdown but eventually we reached shelter and were able to spend a few weeks catching up with family and friends before heading off on the next great adventure to another frozen London, 5000 kms further west, but a place and people that knew how to deal with environmental adversity.

**Figure 21: Dr. John Cassidy Ewart Kaufmann, Chief of Neuropathology, University Hospital, London, Ontario.**
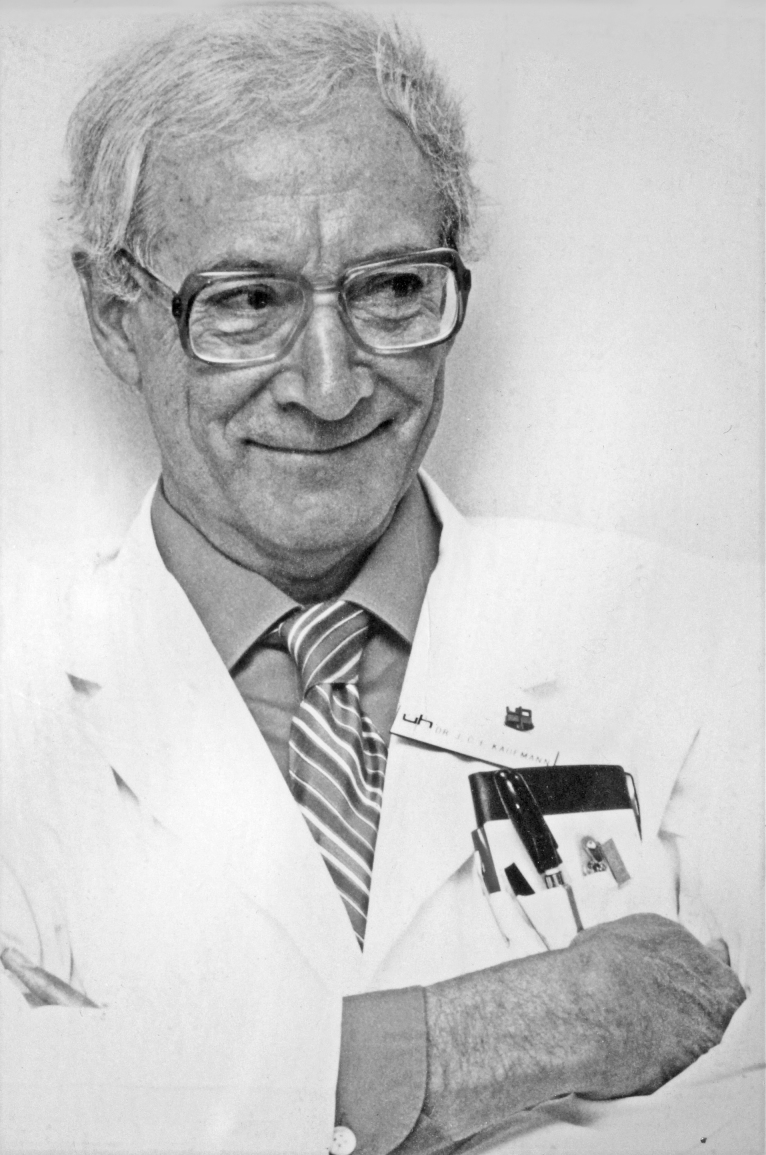


It was decided that I should travel ahead alone to arrange accommodation. I was greeted at London’s small regional airport by my future mentor, teacher and friend, the late John Kaufmann **(Fig. 21)**. Wearing an ankle-length sable fur coat with matching hat, John or JCEK as he was referred to by the residents, warmly welcomed me to ice-bound Canada and promptly brought me home to meet his extraordinary wife Suzanne in whose home I was to remain for over a week as I settled in. I was constantly reminded that I was there as a student of the university to learn. There are lots of hospitals these days, who refer to themselves as University Hospitals, but I am not sure the management of those institutions fully realise what it means to be a true University Hospital.

## London Ontario and neuropathology education

The contrast with the pressures (self-imposed) of the other London receded, as I met the residents in neuropathology as well as the rotating residents from neurology and neurosurgery and from whom I was to learn so much. Quickly, I found an on-campus house that was to remain our cheerful home for the next 3 years. Sandra and Katie soon followed to Canada, a tricky journey carrying a baby plus all the bits and pieces, a journey made worse by having to travel through the UK with an unscheduled stop in Gander before catching the Air Ontario connection in Toronto. The university accommodation campus was home to medical residents from across Canada and the rest of the world, many wishing to spend time in what was then Canada’s leading Clinical Neurosciences department. It was easy for us to form friendships which are as strong today as in those days of study and penury. Charles Drake, Henry Barnett and John Kaufmann led the way in attracting residents to London, but there were so many others working in that fertile environment who helped make Western’s Neuroscience department one of the world’s very best. Neuropathology training was meticulously organised with exacting standards.

I soon realised that having the MRCPath examination was of little value as neuropathology was so different to histopathology. Every autopsy was an educational experience to be shared with others in the form of a weekly clinicopathologic conference (CPC). Preparation for the weekly CPC necessitated reducing the clinical details to a single page of type script with a one-inch margin, which was then given to attendees at the conference. The neuropathology resident who had prepared the clinical summary read out the findings as the clinicians assembled their thoughts, made notes in the margin, and prepared to discuss the case. Beginning with the most junior resident and proceeding upwards in seniority, it was expected that each resident would build on the preceding discussion, following classical localisation rules and conclude with a pathology differential diagnosis. Dare any resident begin a discussion without adhering to the rules! Finally, a consultant who had not seen the case in life would be asked to discuss the diagnostic possibilities before the neuropathology resident revealed all and prepared for incoming fire.

There was a particular consultant who was brilliant in summing up, invariably time and again arriving at the correct diagnosis, much to the annoyance of the neurology residents. He just seemed to have an ability to take all of the resident’s nervous mumblings and draw them together into a coherent intelligent summary. One day, we arranged with the chairperson that the suave consultant would be asked to discuss the case before the residents! Chaos ensued. Tempers were lost. It was the challenging case of a young women who had developed an MCA stroke three weeks after a steering wheel crush injury to the chest. All the usual suspects were rounded up – arterial dissections of several types, paradoxical embolization and so on, but try as he might the now not-so-suave consultant could not explain the unfortunate woman’s death. When I showed that the MCA embolus had originated from a large pulmonary vein thrombosis, he graciously accepted defeat and applauded the neuropathology input to the discussion. Every week it was the same – challenging cases, pin-point diagnoses, and top-class chairmanship and so on. Lasting 3 hours, the weekly neuroscience conference managed to capture and captivate all the of the neuroscience specialties into a harmonious educational experience, which I doubt has ever been surpassed.

**Figure 22: Dr. Joe Gilbert, Chief of Neuropathology, Victoria Hospital, London, Ontario.**
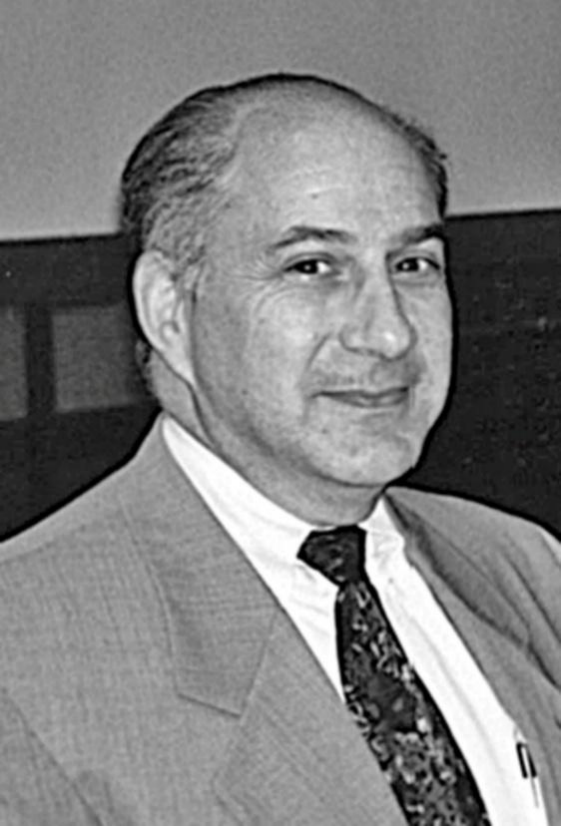


If CPC participation were not enough, the neuropathology residents gathered at an octopus like multiheaded microscope for the weekly slide review. The ten unknown haematoxylin-stained sections, devoid of any history, were left out for a 20-minute review before the Consultant asked each to describe the findings, beginning with the slide topography. Again, the process was repeated, junior resident followed by more senior residents with rotating residents from neurology and neurosurgery treated the same as the hard-core neuropathology residents. Many of the rotators had sharper eyes than the neuropathology residents. As soon as every tiny, microscopic feature was extracted from the slide, the discussants were asked in turn to suggest the clinical findings, in effect a reverse of the earlier CPC. To this day, I still feel that I cannot leave the haematoxylin-stained section without having prepared a differential diagnosis before having the diagnosis refined by immunocytochemistry or genetics or the latest version of a methylation profiler. That tradition which began with John Kaufmann and Joe Gilbert **(Fig. 22)** is today continued at Western by Rob Hammond and team and reaches across the world through internet discussion of digitised slides.

 Western’s autopsy rate was high with brain examination and retention invariably carried out at each autopsy. Weekly brain cutting sessions were legendary – brains were carefully sectioned and laid out on large metal trays by the resident – it didn’t matter whether you were a neurology rotator or a neuropathology resident – high standards were expected. Once the brains were ready, either Joe or John would arrive – the former usually carrying a large mug of coffee – the findings were presented – Joe especially, could reconstruct the clinical story from the brain whereas John was precise in his anatomical localisation and in finding lesions the resident had invariably missed. On one occasion brain slices were transposed so that right was left and vice versa – it was several years before I figured out how Joe could tell left from right! Failure to horizontally transect the mid brain was a terrible crime. The sessions were full of learning anecdotes which I have never forgotten. A few weeks later the slides would come through and each and every slide was examined in detail at the multiheaded microscope, after which reports were prepared and suitable cases were chosen for the weekly CPC often leading to publications **[3,4]**. I am confident that the autopsy surpassed all other learning experiences throughout my career. To witness the current decline in autopsy practice among pathologists and some neuropathologists, is for me a harbinger of the death of neuropathology as a clinical neuroscience specialty. What will remain of our once exciting specialty in which even the pathologist’s opinion of the tumour slide will count for nothing as it is replaced by the dismal dogma of tumour methylation profiling. 

## The AANP and CANP

 From London I travelled to my first meeting of the American Association of Neuropathologists in Philadelphia in 1982 and my first meeting of the Canadian Association of Neuropathologists in Banff in 1983. It was thrilling to witness the greats in action as over the years I watched in fascination as Stan Prusiner advanced the Prion concept in the face of ferocious and often vitriolic opposition. I think back to a patient with a familial cerebellar syndrome I presented at the CANP. Nobody could explain the large multicentric amyloid containing cerebellar plaques. The case was later published in the Annals of Neurology **[5]** where Arthur Hudson raised the possibility that our Italian patient’s familial Gerstmann Sraussler Scheinker disorder might have been caused by consumption of home-bred rabbit! A few years later my great pal Harry Vinters **[6]** and others confirmed the transmissible nature of GSS **[7]** in this family. I have tried to get to the AANP at least every second year as it is the world’s foremost neuropathology meeting. The opportunity to meet friends is a great attraction. The educational programmes run by the AANP both at the annual meeting and on-line are simply the best there is. 

**Figure 23: Professor Mike Schkrum and wife Sue.**
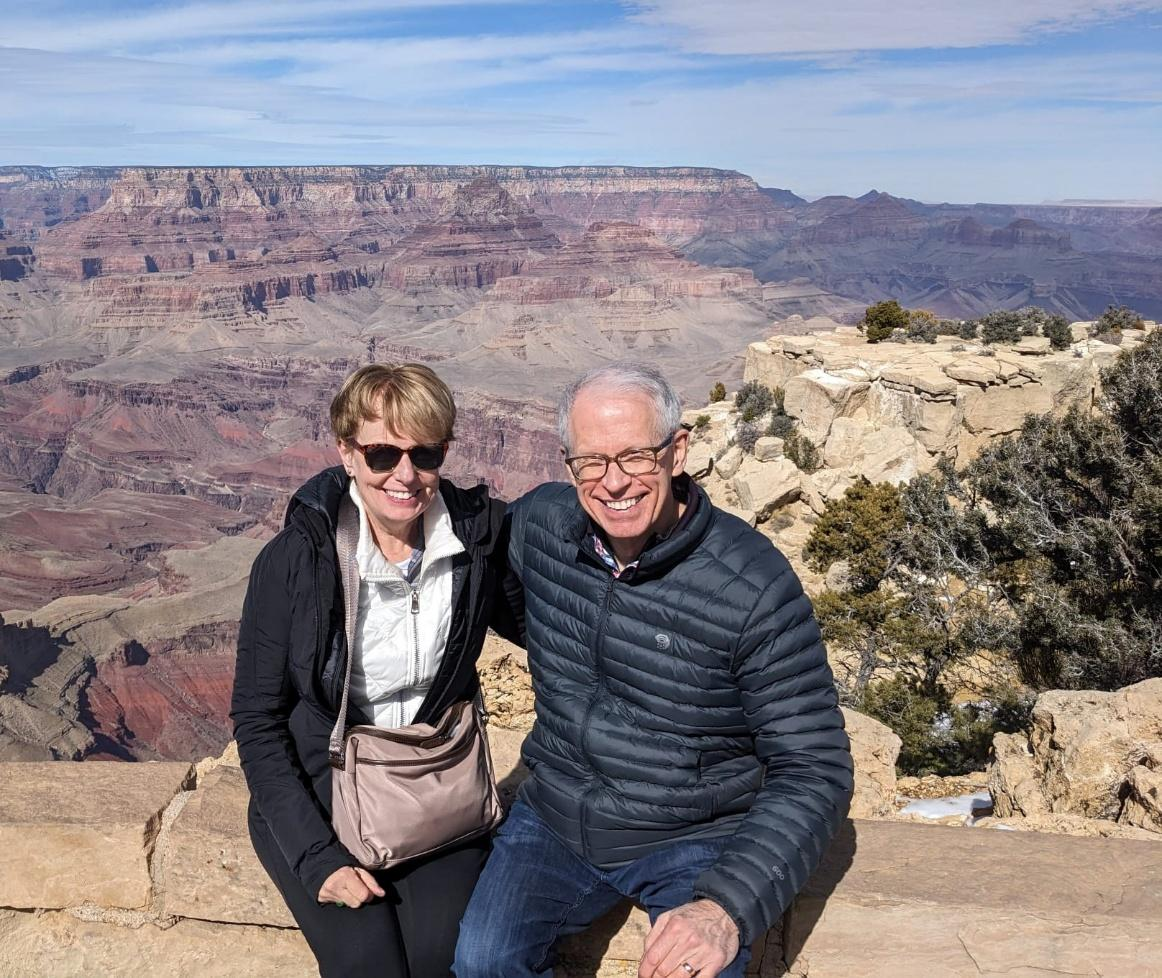


Edward our second child was born in London, Ont. so we were in the unusual position of having two Londoners, albeit born 5000kms apart. We made lifelong friends at Western especially Mike Shkrum, his wife Sue **(Fig. 23) **and their family. Lee Voulters, **(Fig. 24)** an eminent Mississippi Neurologist who later completed fellowship at Columbia with Stanley Fahn is in regular contact to discuss the travails of English Rugby Team. Jon Stoessl has been a world leader in movement disorders and with his wife Cathy has visited Dublin on many occasions. Mark Sadler who led the way in Epilepsy Care on Canada’s east coast sadly died a few months after we dined in Dublin on the eve of the 2023 Rugby World Cup. Although not a resident, Greg Cairncross was a newly arrived neuro-oncologist who along with David McDonald were pioneers in neuro-oncology. It was Greg who laid the ground for 1p19q to become the signature marker of oligodendroglioma. John Noseworthy, a brilliant junior staff neurologist later became CEO of the Mayo Clinic.

**Figure 24: Neuroscience Residents v Staff Neurologists Softball Tournament, Western University 1983. Left to right standing. Peter Gates, Lee Voulters, George Ebers, John Noseworthy, Greg Cairncross, JF Lemieux, Michael Farrell. On ground, not identified at this time, Rick McLachlan and Jon Stoessl.**
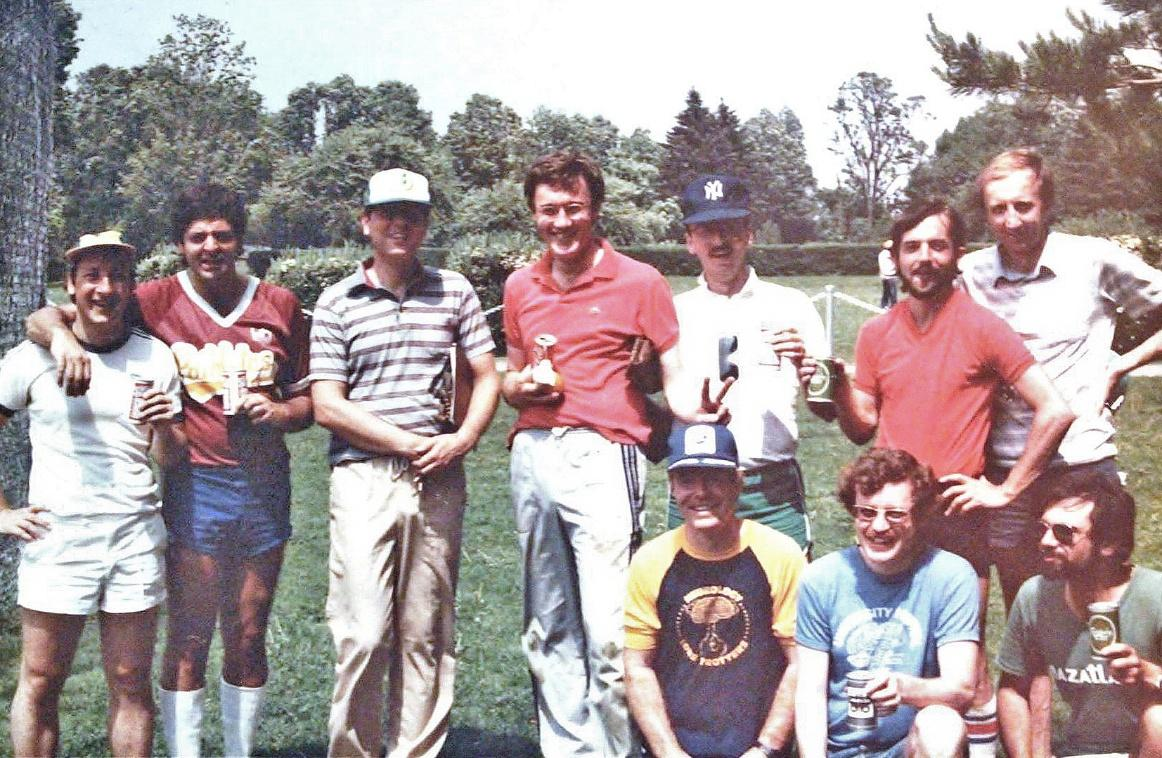


## Dabbling in Neuroimmunology

**Figure 25: George Ebers, Professor Neurology, Western University.**
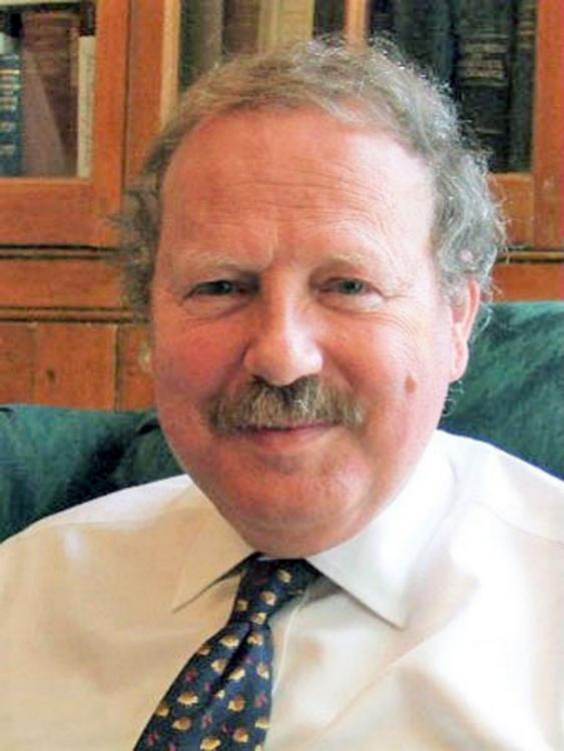


 When my neuropathology residency was completed, I had an opportunity to spend time in George Ebers’ **(Fig. 25)** neuroimmunology laboratory at Western. It was the first time I did any bench work since Minneapolis. Holly Armstrong did her best to teach me her meticulous laboratory techniques. At that time George was the world’s leading expert in multiple sclerosis (MS) genetics and epidemiology. A brilliant Columbia educated neurologist-scientist George was bursting with ideas and enthusiasm. He was responsible for helping many young Canadian neurology residents to have outstanding careers in MS research, most notably George Rice, John Noseworthy and Brian Weinshenker. I was tasked to correlate MS plaque morphology with the presence or absence of oligoclonal bands (OBs) **[8]**. Along the way I learned the techniques involved in CSF analysis for OBs and later with Holly’s invaluable help, I introduced the technique to the Richmond Hospital in Dublin. 

I recall seeing a young woman with opsoclonus -myoclonus whilst on rounds with George. Without even a cursory examination of any kind, George simply requested an abdominal ultrasound which, predictably, found a large ovarian mass. On hearing the ultrasound findings, George correctly predicted there would be brain tissue in the mass. My great friend Mike Shkrum, then a surgical pathology trainee and now one of Canada’s leading forensic pathologists, dissected the ovarian mass and lo and behold here was a large area of perfectly preserved cerebellar cortex in the middle of the ovarian teratoma. Following removal of the teratoma, the patient’s eye signs improved overnight. I was able to show that the patient’s serum reacted with the Purkinje cells in fresh frozen normal cerebellum confirming the presence of anti-Purkinje cell antibodies. Unfortunately, we didn’t have any frozen ovarian teratoma on which to run a western blot. Undaunted George said we should passively transfer the antibody to mice to see if we could generate any neurological signs. I began to inject patient’s serum into the cisterna magna of mice – naturally most of the mice died as a result of my clumsy attempts to locate the tiny cisterna magna – but a few mice survived, and we were able to show that patient’s serum had decorated the mouse Purkinje cells, although none of the mice developed signs of ataxia or opsoclonus. I was disappointed that we never wrote up the case as it was just at the time when neuro-immunology was about to explode.

## Leaving Canada and Harry Vinters

Holidays during our time in Canada involved return visits to Ireland to visit aging parents, Sandra travelled back to Ireland in the summer months with Katie and Edward and myself during the winter. Travel between Ireland and Canada involved a routing through Toronto and London, England, which with two small children was difficult and expensive. Luckily, we also got to spend a sunny June week on Lake Joseph, about 4 hours north of London, Ont. Foolishly, after the long hot drive I plunged into the attractive azure, blue lake only to experience the coldest water of my life!

**Figure 26: Professor Harry V. Vinters, Chief of Neuropathology UCLA.**
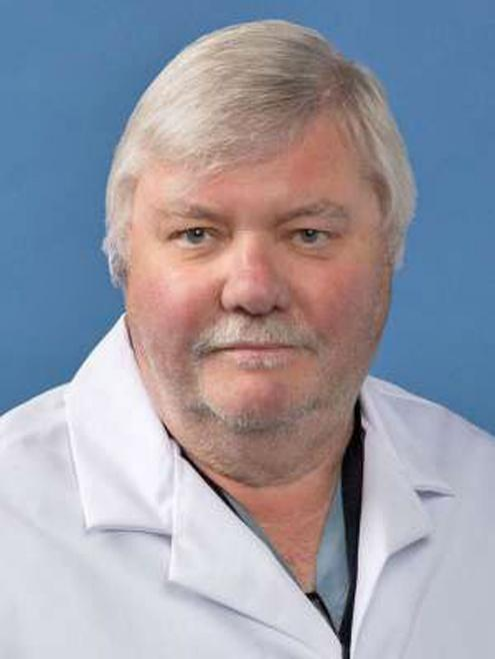


In London, I had heard so much about a former neuropathology resident called Harry Vinters **(Fig. 26)**. Having trained in London, Harry had gone south to work with Pat Cancilla initially at Iowa, but when Pat moved to UCLA as head of pathology, Harry went with him and has remained there as one of, if not the world’s greatest neuropathologist. Harry had promised to return to Western for a year to see if he would like to take up London’s offer as Head of Neuropathology. Our time in London overlapped by 6 months during which a lifetime friendship began and has flourished to today, expanding to involve our families and many friends in Ireland and in the USA but more of that later. Harry had been given the task of house sitting whilst John and Suzanne Kaufmann holidayed in The Cape. Their magnificent residence on Gloucester Road was a treasure trove of African Art, had a large swimming pool and a collection of the best South African wines. On my last day in Canada, Harry threw a party in the Kaufmann residence. It is difficult to recall, but I think there were only three at the party – the wine cellar as attacked with gusto – how we didn’t drown in the pool I will never know – the African art remained intact – the journey home remains a blur, and I know that Harry had a lot of explaining to do when John and Suzanne returned. And so, the best educational time of my life came to an end as in the mid-1980s I joined the work force of a country on its knees and watched as thousands of the Ireland’s best educated emigrated to Australia, USA and Canada. Were Sandra and I out of our minds?

## Return to Ireland in the dismal 1980s

The 1980s in Ireland was defined by economic hardship and political turmoil but nothing could have prepared me for what lay ahead. The Richmond Hospital, which was my alma mater and that of my father, was in the last stages of its existence. A new hospital was nearing completion beside Dublin’s airport and would replace both Richmond and Jervis St Hospitals, the principal teaching hospitals of my medical school. At that time neurosurgery was also practiced on the Southside of Dublin at St. Vincent’s Hospital. John Dinn, an American born Trinity Graduate, was the attending neuropathologist. John also worked in Trinity Medical School where he was an outstanding researcher especially in the area of B12 deficiency. Additionally, John worked at the Richmond but the demands of being in three different locations were great. I was full time at the Richmond with several hundred neuro-autopsies each year plus a heavy surgical pathology workload. Lab space was shared with Histopathology – there were no dedicated neuropathology lab staff. I didn’t even have a microscope when I returned to Dublin other than a monocular brass microscope which had somehow escaped from the Pathology Museum. All monies were being set aside to commission the new Beaumont hospital whose opening was “imminent”. Anyone with a brain could see that the country was penniless. The new hospital lay complete but empty for three years until finally in late summer 1987, the government announced the hospital doors would open in November. By then I had become so disillusioned that I returned to London, Ontario and worked as locum neuropathologist for the summer of 1986. I had already criss-crossed the North Atlantic to complete both parts of the Canadian Neuropathology Fellowship and so the temptation to accept a permanent contract in London was great. But my wife Sandra was not for moving – the children were already settled in school in Dublin.

## Early days in Temporal Lobe Epilepsy Surgery

**Figure 27: Professor Orla Hardiman, Trinity College Dublin.**
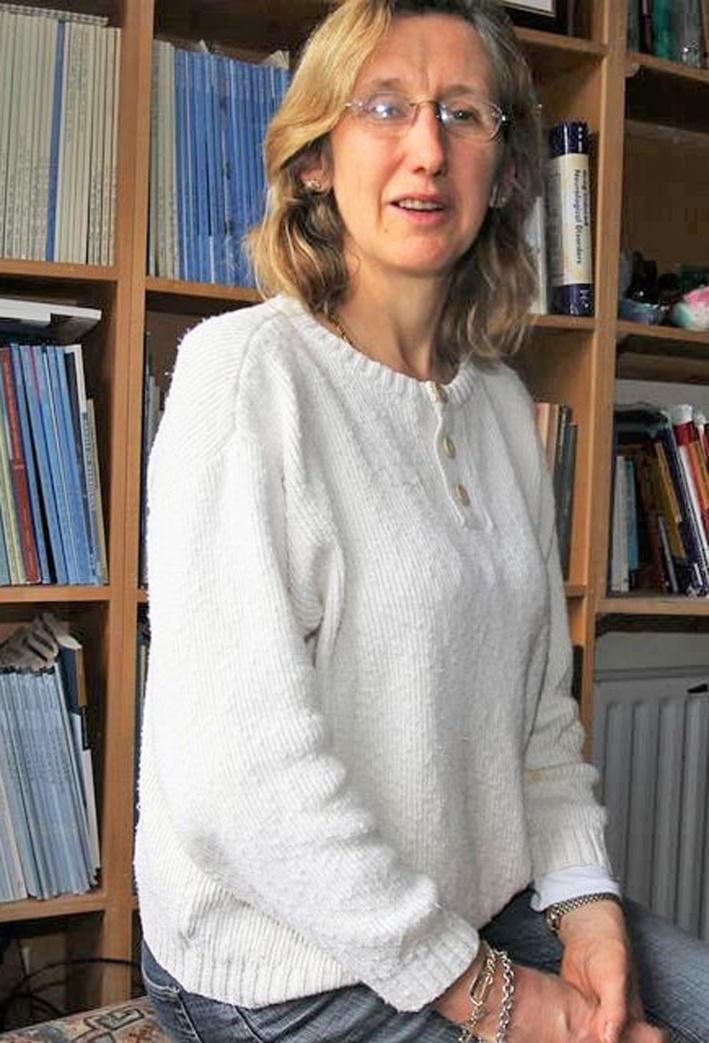


**Figure 28: Dr. Hugh Staunton, Neurologist, Richmond and Beaumont Hospitals, Dublin.**
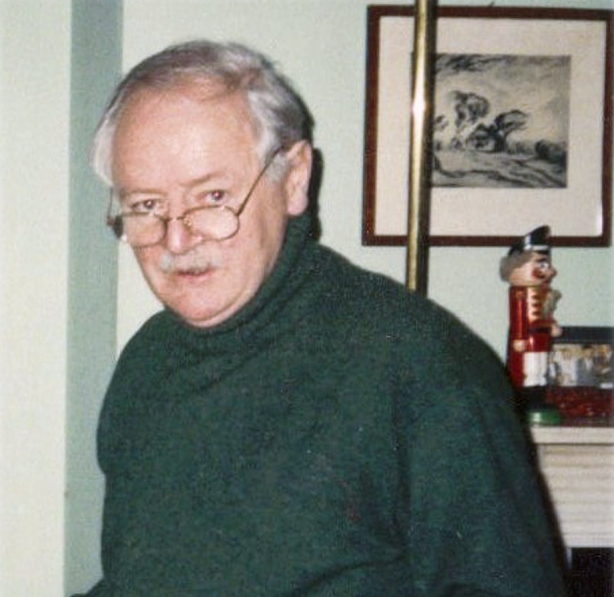


**Figure 29: Jack Phillips, Neurosurgeon, Richmond and Beaumont Hospitals Dublin.**
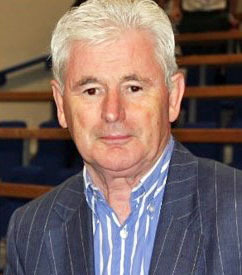


 The years 1984 to 1987 had not been all bad. A brilliant neurology trainee came to spend a year with me in Neuropathology and threw herself into work and research. Her name was Orla Hardiman **(Fig. 27)**, now one of the world’s leading experts in motor neurone disease. Knowing I was interested in epilepsy neuropathology, Hugh Staunton **(Fig. 28)**, the epilepsy neurologist and neurosurgeon Jack Phillips **(Fig. 29)** had retained approximately 50 temporal lobectomy specimens to await my examination as soon as I came back from Canada. The epilepsy surgery programme was in its infancy. MRI was not then available in Ireland, so the resections were carried out on the basis of failed anti-epilepsy medication, EEG localisation, a successful Wada Test and non-lesional imaging as assessed by CT. The patients had all done well following temporal lobectomy. Orla and I ploughed through the archived specimens and immediately set about trying get a handle of microdysgenesis **[9]** or what we now classify according to the ILEA and Palmini criteria. All went well, until a few weeks into the project when it dawned on us that none of the resections included a hippocampus! A full temporal lobectomy was supposed to have been carried out. Hugh Staunton’s face paled when I told him of the missing hippocampi. By then MRI was available across Europe and many of the patients were sent to Germany for post resection imaging and sure enough, the hippocampi were located, still in the patient’s brains! Amazingly the patients had done as well as others from London UK and Montreal in terms of seizure control. The superficial neocorticectomy or the “Dublin operation” as it was called by some and disparagingly referred to as the “Dublin Topectomy” (as in the top of an egg) by others seemed to have achieved the same outcome as both selective hippocampectomy and the full Murray-Falconer type complete temporal lobectomy. The patients did well, not only in terms of seizure control but importantly in terms of reduced psychological deficit with these favourable outcomes maintained for several years **[10]**. Gradually however, the seizures returned and many of the patients underwent repeat surgery to remove the residual hippocampus, usually with a sustained improvement in outcome thereafter. 

## An unhappy transfer to Beaumont

**Figure 30: Dr. Katie Keohane, Neuropathologist, Cork University Hospital, Cork.**
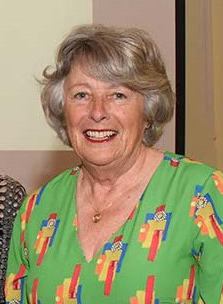


Closure of the Richmond Hospital and transfer to Beaumont was eventually completed in the rain and freezing wind of November 1987. I made several trips on the back of open topped trucks to help safeguard the relocation of valuable laboratory equipment to a new home. The story of the actual moving day is replete with incident but is better saved for a different forum. The staff of the two merging hospitals got to know each other and began to learn how to work together. But there was trouble on the horizon. Tensions were rising over control and future direction of the neurosurgery department and eventually spilled over in public acrimony where charge was followed by counter charge. Many staff who had nothing to do with neurosurgery took sides and, as a consequence, friendships were sundered and hospital efficiency declined. Given that many of the charges involved cases in which neuropathology findings were pivotal, I was drawn into the fray having to answer questions about biopsy and autopsy results. It was an extraordinarily stressful time for all. During the upheaval my father had died in the neurosurgical unit from a ruptured middle cerebral artery aneurysm. The aneurysm clipping was successful but unfortunately the consequences of life-long smoking were reflected in post-operative declining respiratory function and death. Eventually, I had enough and through the kind support of colleagues in histopathology and Katie Keohane **(Fig. 30)**, Cork’s most famous neuropathologist, the hospital CEO granted me leave of absence.

## Sabbatical in UCLA

 Thus, in January 1991, leaving Sandra, Edward and Katie behind, I headed for UCLA where I spent 6 months working with Harry Vinters. The work was long and hard but productive. I learned much in those months. In my absence, Katie Keohane flew up to Dublin from Cork each Thursday, reviewed the week’s cases, both surgical and autopsy and attended the Friday morning MDT before returning, exhausted, to her family in Cork. That winter on the US west coast was the wettest on record. My apartment flooded as overloaded pipes and gutters burst. Harry and I had a memorable drive up the Pacific Coast Highway to San Francisco stopping off to swim in the freezing Pacific. The Rory Gallagher concert at San Francisco’s Stone Club on March 16^th^, 1991, was the highlight of a memorable St. Patrick’s weekend. Working with Harry Vinters was exactly the boost I needed to overcome the hazards of single-handed practice in Dublin. During my time in UCLA, hemispherectomy for intractable childhood epilepsy was really getting into its stride led by outstanding neurosurgeons Warwick Peacock and Gary Mathern. I was privileged to be in a position to review the neuropathology of those valuable and rare resection specimens **[11,12,13]**. We could see the resemblance between the dysplastic cortex and similar balloon cells in tubers resected from children with tuberous sclerosis. We prepared a beautiful poster for the AANP June meeting in which we compared the ultrastructural and genetic features (loss of heterozygosity) of a tuber and cortical dysplasia, which drew the quick comment from a passing esteemed member of the AANP *“Ah – here’s Paddy with his Potato!”.* Popular then was the idea that Rasmussen’s Encephalitis was caused by cytomegalovirus infection **[14]**. In fact, the story had emanated from our old Alma Mater at Western and so it was with reluctance that Harry and I showed that CMV was not the cause of Rasmussen’s **[15]**. I was also fortunate to see at first hand the ravages wreaked by HIV infection on the nervous system along the West Coast and this helped greatly as back in Dublin, Elaine Kay and I finished the work started by John Dinn on Irish patients with fatal HIV infection **[16] **and later managed to expand the work, a little **[17]**. 

## Return to Dublin

Months later I was back in Dublin, reinvigorated and full of work ideas. The Irish economy was slowly emerging from the dismal 80s, although not improving quickly enough to encourage young emigrants to return. Even though Beaumont was located on the far side of Dublin, Sandra and I remained living on Dublin’s Southside. Edward and Katie were attending good schools; their cousins lived around the corner and Dublin’s universities were within walking distance. We were fortunate to live where we have done since 1984 and remain there to this day. As turmoil in the new hospital abated, I began to set about trying to build a department which had the patient at its centre. Fortunately, I had superb laboratory and administrative staff all of whom were focused on generating fast accurate diagnoses and who were led by Maureen Burke, Josephine Heffernan, Olivia Droogan, Caroline Stanley and Carolyn Plummer. The diagnostic surgical workload was heavy, and we did not have the safety parachute of rapid immunohistochemistry, being limited to GFAP and a few lympho-epithelial markers. Molecular diagnostics were years away, but we managed. Digital radiology was also in the future so there were daily visits to neuroradiology where I had many fruitful discussions with outstanding colleagues. A weekly brain tumour review meeting was introduced and became the forerunner of today’s Beaumont neuro-oncology MDT. Back in the early 90s it was always a late Thursday evening scramble to retrieve the Kodachrome slides from the commercial photography lab in time for the Friday morning meeting. I was taught many times by John Kaufman at Western that clinicians had little tolerance for pink microscopic images and to limit my slides to a maximum of three, advice that still holds true even in this digital age. There were about 300 autopsies each year of which a majority had a neuro component. If the patient had been cared for on a neuro ward, I did the complete autopsy otherwise it was brain and spinal cord only. Brain examinations were held on a Wednesday morning and usually attended by interested neurosurgeons and neurologists. One of the saddest things I have lived to witness has been the decline in autopsy work, a decline facilitated by pathologists and indeed by some neuropathologists, who now see themselves as pure tumour pathologists. Pathology was never meant to be solely about oncology. I can honestly say I learned most about medicine from the thousands of autopsies I carried out over almost 50 years of pathology practice. Recently the British Neuropathology Society decided that trainee neuropathologists would no longer be required to carry out full body post-mortem training, as if a stroke autopsy could be meaningfully carried out without examination of the cardiovascular system! The expertise that neuropathologists brought to forensic work will decline. I was very fortunate in being able to have a neurology or neurosurgery trainees rotate through neuropathology. The majority were highly motivated, enthusiastic and prepared to work hard, not shirking at having to do autopsy work or cut-ups. Many are now consultants in their respective neuroscience disciplines. What neurology trainee would today wish to waste time learning the intricacies of glioblastoma subtyping? As I had been thought at Western, interdisciplinary exchange of ideas was what made the neurosciences so special and exciting.

## Neurologic observations

 Single handed practice was busy but rewarding and I was fortunate to have a few willing pathology colleagues who were prepared to look at frozen sections whenever I was out of the lab teaching, attending inquests or on annual leave. I was able to continue some collaborative work on Rasmussen’s with Harry Vinters **[18,19]**. Encouraging the clinical rotators to produce at least one publication during their spell in neuropathology led to some very interesting case reports. I remember a young woman who presented with a seizure and was found to have a large ring enhancing temporal lobe lesion that the surgeon *“scooped out with his index finger”* and which turned out to be acute tumoural multiple sclerosis. That in itself was mildly interesting, but I was later told the patient had received a sibling bone marrow donation from a brother for treatment of childhood leukaemia some 20 years previously. My great colleague Hugh Staunton and I set about trying to prove that the participating T-lymphocytes in her MS plaque were from the patient’s brothers. Sure, enough with the trans- Atlantic collaboration of Elizabeth Unger, the lymphocytes labelled with a Y-Chromosome marker proving their fraternal origin. That wasn’t the end of the story. The donating brother was imaged, and multiple clinically silent demyelinating plaques were identified on his MR, good evidence of passive MS transmission. The sibling’s demyelinating condition went on to behave in every way as relapsing – remitting MS and not as a familial leukodystrophy. Hugh and I always felt that the case never really received the credit it deserved even when published in a very prestigious journal **[20]**. 

## A colleague arrives via San Diego

**Figure 31: Dr. Francesca M. Brett, Neuropathologist, Beaumont Hospital, Dublin.**
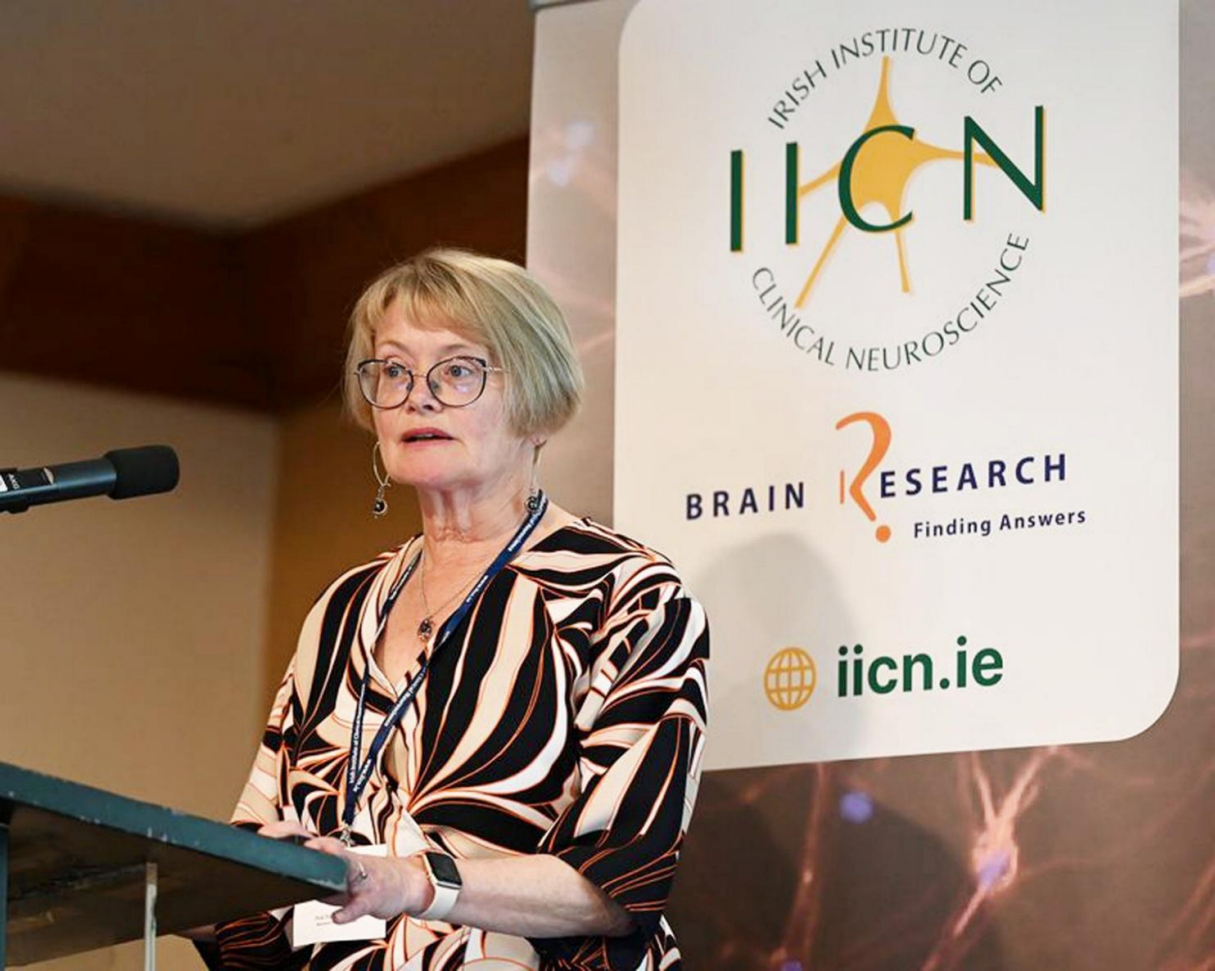


 This was the busiest time of my life, and I was finding it increasingly difficult to stay abreast of the workload. John Dinn sadly died a few years earlier. I persuaded the hospital to source funds for a second neuropathologist, and in 1995, Dr Francesca Brett **(Fig. 31)** fought off stiff competition for the position of neuropathologist at Beaumont and at Trinity College Medical School. I thought all my prayers had been answered as Francesca had been working in Cambridge University with John Xuereb and Janice Anderson and was clearly well trained and well suited to the rigours of Irish Neuropathology. But there was a hiccup in that the external interviewer, the great Dr. Harry Powell from San Diego immediately invited Francesca to spend a few months in his laboratory at UCSD. I could hardly say no, and so Francesca flew off to the West Coast where she immersed herself in American Neuropathology learning so much from Harry Powell and Larry Hansen. Like my time at UCLA, Francesca’s time at UCSD translated into ambition and high standards which were maintained at Beaumont for many years thereafter. Our interests back in Dublin were broadly similar and as the publications have shown, were focussed on good quality clinical neuroscience in its broadest terms **[21,22,23]**. Francesca also developed expertise in Forensic Neuropathology and is today the “go to neuropathologist” on matters forensic in Ireland. I continued with my interest in epilepsy neuropathology **[24]** and eventually managed to make it into the Journal of Neuroscience **[26]**. 

## Landmarks in Neuropathology

In more recent years, I suppose the really big events in neuropathology were variant CJD, Traumatic Encephalopathy and DNA Methylation Profiling. I had paid little attention to the unfolding crisis in the UK Beef industry as more and more cases of Bovine Spongiform Encephalopathy were reported across UK farms through the late 1980s. Even when the bovine brains were shown to exhibit the same spongiform change as ovine scrapie, there were constant reassurances that as sheep were safe for human consumption, even when infected with scrapie, there was no need to worry that BSE might cross the species barrier. The death of two farmers from CJD on England’s South Coast was not a cause for concern. But bowing to increasing public concerns about the safety of British beef, it was agreed that all brain and spinal tissue should be removed from bovine carcases before processing for human consumption. As was so eloquently set out years later in the 2000 Phillips report into the BSE– CJD scandal, blame was apportioned to government departments, committees and systems of communication that had sought to protect the British billion-pound beef industry. Nine years after the emergence of BSE, James Ironside and Bob Will reported the first case of variant CJD with its unique neuropathologic change. It wasn’t long until the funeral pyres of incinerated cattle were visible from space.

## Variant CJD in the Republic of Ireland and tonsillectomy

Needless to say, the Republic of Ireland with the second highest incidence of BSE worldwide waited with nervous anticipation for its first case of vCJD. By 2003, only a single case of vCJD had been identified in the Republic with only another three cases to follow. The index case was a young woman with recent onset of ataxia who was discussed at the Beaumont Hospital Friday morning neuroscience meeting. There was no family history of ataxia. Drugs and alcohol were excluded. There was no underlying malignancy. When someone from the rear of the lecture theatre innocently suggested variant CJD as a possible diagnosis, you could have heard a pin drop. Over the next few months, the patient deteriorated and died with the autopsy confirming vCJD. In terms of risk factors the patient had lived in the UK throughout the height of the BSE crisis, a fact that was used by the Irish Department of Agriculture to reassure all that vCJD was a UK disease. Unfortunately, the patient had undergone an endoscopy during investigation of the ataxia. The endoscope was one of five endoscopes later used in just under 50 patents. Not knowing much about the transmissions risk of gastrointestinal tissue or which of the five endoscopes was used it was decided that all of endoscopes should be destroyed and that the 50 or so contacts would be advised of the transmission risk, on the basis of full and open disclosure. None of us had any notion about the transmission risks of endoscopy. Yet, we had to provide some idea of risk to the 50 patients – this was simply not possible and many of the 50 who were informed suffered tremendous anxiety for years afterwards. It was yet another example of the inability of full and open transparency to convey risk – once a risk is perceived, no matter how small, it is still, in the eyes of the public, an anxiety provoking risk. The activist can always point to a risk whereas the expert can never deny the possibility of risk and so loses the argument. Doorstepped outside the hospital I was interviewed on national TV about transmission risks – I did my best to reassure the public but don’t believe I was successful. Later while being interviewed on morning radio, I used the analogy of trying to detect the remains of a drop of coloured water released into a large Irish lake, but again to little avail.

**Figure 32: Professor Michael Walsh, Professor of Otorhinolaryngology RCSI and Beaumont Hospital, Dublin.**
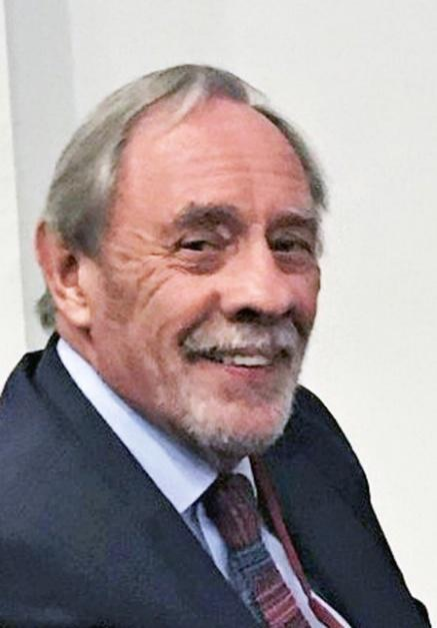


**Figure 33: Dr. Rachel Howley, Senior Scientist, Beaumont Hospital Neuropathology.**
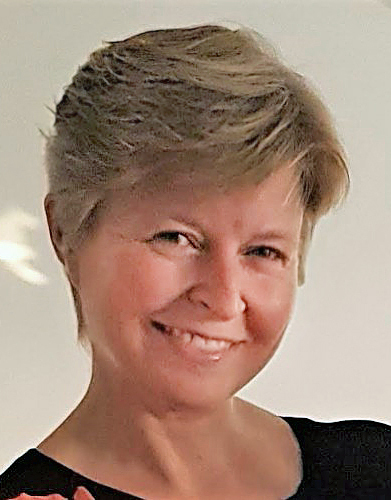


 Meanwhile Ireland’s then Minister for Health and current Taoiseach, Michael Martin was concerned about vCJD transmission during tonsillectomy and so I was summoned to the Department of Health to discuss the introduction of disposable surgical instruments for tonsillectomy. Prior to the meeting I met my good friend and outstanding ENT surgeon Professor Michael Walsh **(Fig. 32)** who gave me a live demonstration of tonsillectomy. I was horrified at the procedure’s difficulty and the potential for blood loss. Meeting the Minister and his officials, Michael Walsh and I took the line that it would be a mistake to introduce disposable instruments **[29]**. The fact that we saved the Department of Health around 30 million euro was secondary to the fact that at least two individuals undergoing tonsillectomy in another country died following the use of disposable instruments. One remote risk had been replaced by a real and substantive risk of immediate death. An upside of the crisis was our ability to obtain funding for development of a top-class National CJD Surveillance programme led by Rachel Howley **(Fig. 33)**. Western Blots and RTQuIC were established by Rachel who also managed to complete her PhD on Glioma Biology while working as a scientist in the lab. Rachel has green fingers – there is nothing she can’t do in the lab or outside. Building a house extension – remodelling her camper van or sequencing mitochondrial DNA – are all achieved with ease. 

## A catastrophic event

 Ten years later I was again confronted by an even more immediate problem of risk assessment and conveyance. A young man underwent emergency partial hemispherectomy for intractable epilepsy, which had progressed to probable fatal status epilepticus. Surgery was considered the only option to save his life. The resection was carried out on a Thursday during which I had been requested for an intraoperative frozen section. On Friday I scanned the tissue sections and all I could see was cortical gliosis and a meningeal – dural cicatrix which related back to a penetrating head injury some 30 years previously, when as a young boy, the patient had been involved in a farmyard accident. In addition to cortical gliosis, there was vacuolar change which I mistakenly attributed to status epilepticus. Just to be sure though, I asked Ciara, one of our outstanding histotechnologists if she would do a 3F4 Prion stain. *“No problem”,* replied Ciara, *“I will have it for you on Monday morning*”. Interpreting 3F4 immunostaining can be difficult, but there was little doubt when I saw the stained sections on Monday. The controls were satisfactory. My sense of impending doom quickened and reluctant to believe what I was seeing; I asked Ciara to repeat the procedure. The result was the same, just more convincing. I signed out the report of CJD, alerted my colleagues in Infectious Disease - Microbiology and most importantly alerted the neurosurgical operating room only to discover that the surgical instruments had already gone back into circulation. A high-level emergency team was assembled and so began at least 3 weeks of non-stop frenetic activity, which involved the termination of all neurosurgical activity and tracing of all patients who might have come into contact with infected instruments. We located the patient’s operative notes from his craniotomy 30 years previously, and sure enough, in beautiful copper plate writing, there was the surgeon’s description of Lyodura insertion to cover the penetrating dural defect. Risk calculations were attempted based on whether the exposed patients had undergone penetrating brain procedures or not. Outside assistance and advice were sought from James Ironside and from the great Paul Brown at the NIH. Paul sadly died in August 2025. Their help, reassurances and advice were invaluable. The risk committee led by Paul Brennan (an interventional neuroradiologist and real “doer”) managed to handle the situation in the face of understandable public concern, and within weeks risk had been conveyed to all known suspects **[30,31]**. For some, it didn’t matter as they were already suffering life changing fatal brain tumours. For many others with normal life expectancy, the future would always be associated with risk, reinforced by the knowledge they would be unable to donate blood and would always be regarded themselves as a “CJD Risk Patient”. 

## The happier side of CJD

The “CJD Period” was greatly enhanced and indeed enlightened by the annual EUROCJD meetings hosted by Herbert Budka in Vienna. For me, these meetings were the highlight of the academic year. Outstanding speakers such as Adriano Aguzzi and Bob Will were invited. Meetings were held in magnificent venues around Vienna and were invariably topped off by wonderful dinners and, of course, lots of local Gluhwein, of which Herbert was a connoisseur. Walks through the Vienna Woods were conducted by Herbert who became emotional on recounting the tragic deaths in early 1889, of Crown Prince Rudolf and his mistress, 17-year-old Baroness Mary Vetsera. As Rudolf did not have a son, succession passed to Archduke Karl Ludwig and his eldest son, Archduke Ferdinand whose assassination in June 1914 precipitated the July crisis and the beginning of World War 1.

**Figure 34: Dr. Pat Wallace and Partner, The late Siobhan Cuffe.**
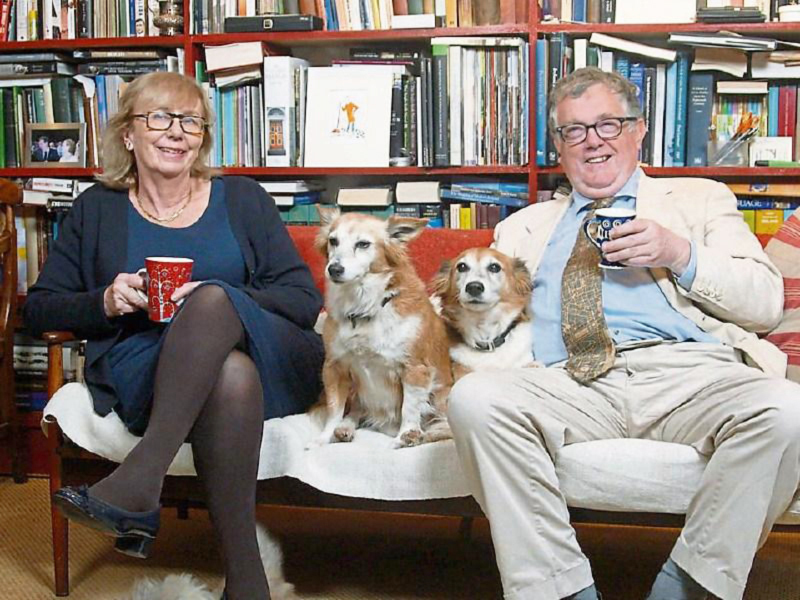


**Figure 35: John McGahern, Author.**
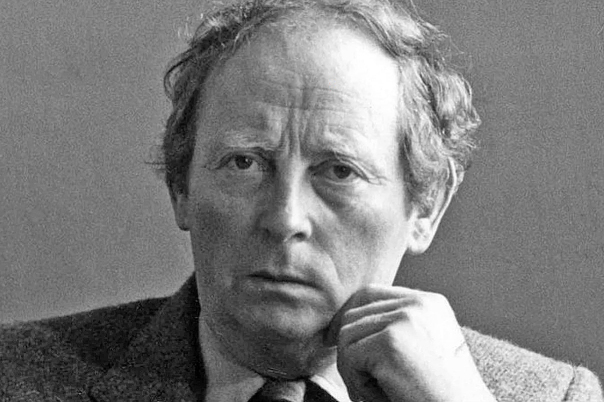


In the middle of the CJD crisis I was elected President of the RCSI Biological Society and one of my duties was to deliver the Presidential address. Needless to say, I spoke about the incredible history of the CJD, paying tribute to my heroes Stan Prusiner and Bill Hadlow. A huge attendance was present, not to hear me, but to listen in awe as my two guests spoke. Dr. Pat Wallace, **(Fig. 34)** the Director of Ireland’s National Museum, spoke about the importance of the cow in Ireland’s long history, whilst Ireland’s greatest living writer and fellow Leitrim man John McGahern **(Fig. 35)**, held the audience enthralled as he discussed the cow in Irish Literature. It was one of the greatest nights in the history of the Bi-Soc since it was founded in 1932!

## Another Neuropathology crisis

 Not long after epidemiology studies showed that there were few if any new cases of vCJD, another neuropathology epic came to international prominence and remains there today. Chronic Traumatic Encephalopathy (CTE) had been recognised as far back as the 1920s and was brilliantly described by Nick Corsellis and Clive Bruton in their seminal 1973 paper "The Aftermath of Boxing". That publication **[32,33]** became the motivation for changes in boxing legislation, most notably the reduction of rounds in world championships from 15 to 12, the compulsory use of headgear in all amateur contests and the total abolition of boxing in all UK schools. I was fortunate to meet Clive on several occasions. He was a frequent visitor to Ireland where he indulged his interest in horseracing. So, with that background I couldn’t understand the fuss that was being made about CTE. What was new? Nick and Clive had said it all. However, once the possible long-term risks of developing CTE from contact sports especially from American Football and Rugby were aired in the public press and on screen, the runaway train could not be stopped. There was and will always remain the possibility of risk for developing a neurodegenerative disorder, not just CTE, from any contact sport. Following on from the work of Nick and Clive, risk modification was studied and applied to all contact sports. Eventually there comes a point where any further attempt at risk reduction renders the sport sterile and devoid of participant or spectator reward. To suggest that parents who allow their children play contact sport are guilty of a form of child abuse is itself an intolerable abuse of science. Guns and cars are not banned because they cause brain damage and death. I played rugby badly and sustained head knocks, one of which resulted in a severe concussion for which I was detained overnight in hospital, but I have no regrets whatsoever nor did I or do I worry about my son or grandson playing rugby. 

 As a rugby fan I was alerted one day when a request came through for an autopsy on a middle-aged man with atypical Parkinsonism. The deceased patient’s name sounded familiar, but I said nothing and just got on with the autopsy. In advance of seeing the slides but knowing the patients background, I requested tau immunostaining on several blocks. The slides came through and I didn’t have to look far to see there was extensive perivascular and astrocytic tau deposition in the sulcal depths. There wasn’t a Lewy body in sight! I called my colleague Willie Stewart in Glasgow who flew to Dublin next morning and independently examined the slides. I can still see Willie coming out of the reporting room and nodding in assent *– “Yes this is definite CTE”*. As we later reported **[34]**, the deceased patient was a top-class rugby player who was at least of international standard and should have been selected for Ireland. Back in those days when the Irish Rugby Team was not up to much, it was harder to get off the team than be selected to play and it certainly didn’t help if you were perceived as being a robust player. Maybe Ireland needed more players like our unfortunate patient. 

## Dementia risk and CTE

 I have been involved in lots of CTE work since we published that case **[35]**. I try to avoid public commentary. I feel strongly that lives are not meant to be lived worrying about remote risks. Right now, it is simply not possible to provide any meaningful risk assessment of the probability of developing any neurodegenerative disorder as a result of concussive brain injury. What is urgently needed right now in all contact sports, is an objective non-clinical measure of concussion severity, duration and reversibility. Data generated by instrumented mouth guards (iMG) are emerging and beginning to show that Peak Power which reflects the severity of a head acceleration event (HAE) is the best measure of the rate of change of kinetic energy that the head undergoes during the acceleration – deceleration which causes the concussion. Of course, in isolation, iMG generated data will only provide a snapshot of the actual concussive event. But if that acute, once-off data could be married to an objective biologic measure of the degree of axonal stretching which underpinned the concussive event, then a simple biologic marker could be used to track the time taken for return of axonal function to persistent normality. A blood test, which measured the degree of axonal stretching as determined by the iMG could be repeated on several occasions, until the test returned to normality after which the player could return to play. Slow return to normality or failure to return to normality as measured against each player’s pre-season baseline blood level would be the gold standard and would, along with measurement of additional brain biomarkers facilitate long term follow up and diagnosis of the onset of neurodegeneration. 

Blood biomarkers for diagnosis of Alzheimer disease and for measuring the rate of axonal loss in multiple sclerosis are already available. Measurements of plasma neurofilament light chain have already proven useful in the assessment of retired rugby players and of childhood concussions. Availability of a blood test which accurately reflected all aspects of concussion would open the possibility of medical interventions to reverse the change, possibly through use of anti-inflammatory agents that might curb the influence of activated microglia.

In rugby, the time has come to link on-field iMG data with off-field biologic data which can provide an independent assessment of the stretched nerve fibre’s response to injury. All that is needed are players willing to participate by providing a blood sample immediately after a game-ending concussion plus a few follow-up blood samples taken over a 2–3-week period followed thereafter by a yearly cognitive assessment with blood sampling.

**Figure 36: The Hospital Pass Front Cover.**
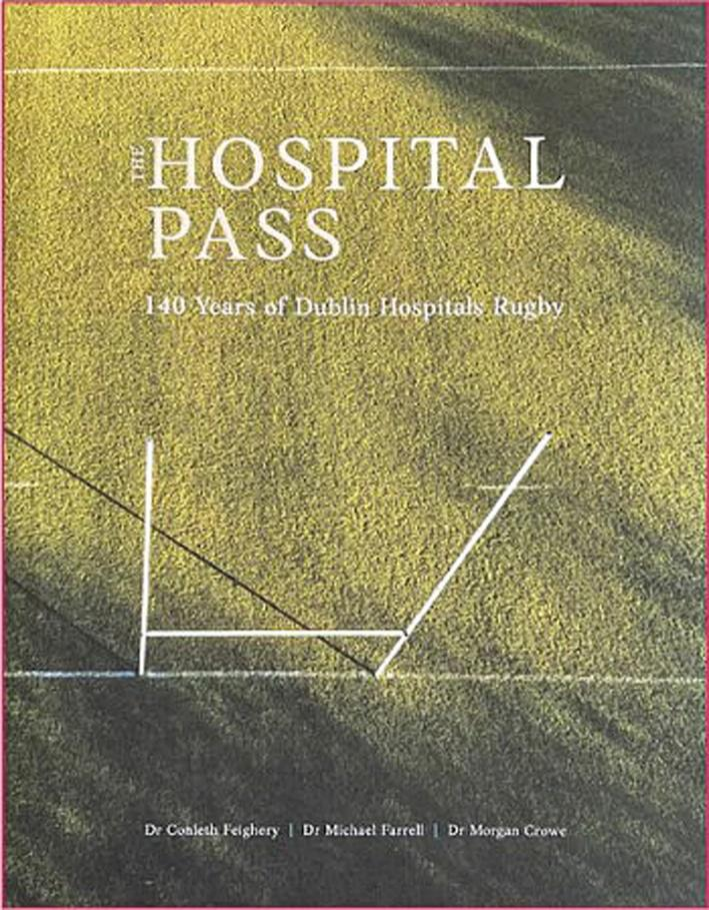


In Ireland and Dublin in particular, rugby has played huge part in the lives of medical students. Across Ireland, more than 200 medical students and graduates, both men and women have been selected to play for the National Irish Team. In Dublin, the inter-hospital annual rugby competition has been ferociously contested for over 140 years. The magnificent silver cup awarded to the winning team is the oldest existing trophy in world rugby. Drs Morgan Crowe, Con Feighery and I have published a history of this great competition **(Fig. 36)**.

## The molecular era arrives

**Figure 37: Dr. Patrick Buckley, Geneticist, Neuropathology, Beaumont Hospital.**
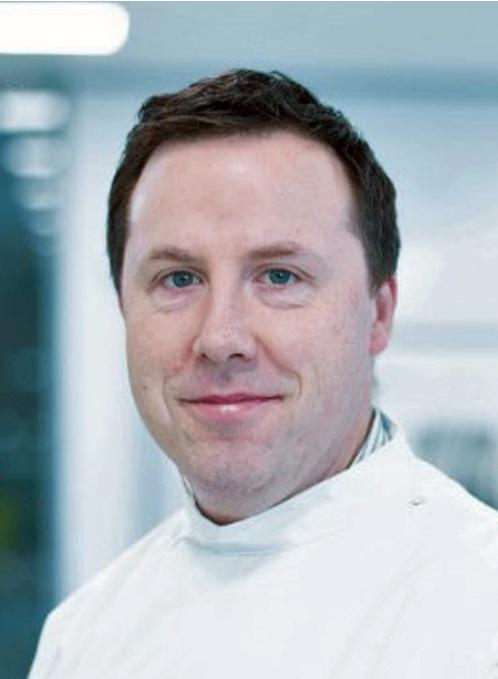


 A few years prior to formal retirement in 2015 and aware of the many developments in neuro-oncology, Francesca Brett and I managed to convince the hospital of the need to appoint a geneticist to neuropathology. Within a few months of Patrick Buckley’s **(Fig. 37)** arrival, he had set up the MGMT **[39] **assay and moved the 1p19q testing to array CGH. Several years prior, Christine Fuller arrived from Rochester and set up FISH for us in the routine neuropathology lab. Josie Heffernan produced excellent FISH images and high-quality results, which even today have stood the test of time when compared with any repeat aCGH testing. But FISH was hard work. Patrick also introduced single mutation testing but, more importantly, began to build a molecular neuropathology laboratory, recruiting and training staff whilst also finding time to become a fellow of the Royal College of Pathologists though his published work. It proved impossible to hold on to Patrick and soon he moved on to establish an incredibly successful independent Genetics Laboratory. He remains an important figure in neuropathology and is very proud of his input to the work that led by his protégé, Teresa Loftus to build Ireland’s most modern molecular pathology laboratory in Beaumont. My colleagues and I were among the numerous authors **[40]** on the landmark study that established DNA Methylation profiling as a core investigation in Neuro-Oncology. The last 8 years have seen an explosion in its application almost to the point where clinicians no longer accept an opinion derived from a slide diagnosis. Neuropathologists have become afraid to commit to a diagnosis without the safety harness of molecular diagnostics. Additionally, publication of the newly revised WHO Classification of Brain Tumours in 2021 so soon after the 2016 WHO Classification, whilst a huge achievement, has created difficulty. Clinicians have been overwhelmed by the many new tumour types and are becoming increasingly frustrated at the 2 to 3 weeks wait required for the combined integrated diagnosis to be released. There is huge uncertainly about the natural history of many of the new tumour types and a persisting realisation that the list of available therapeutic options for adult patients has not really lengthened. Of course, there have been spectacular advances in the management of childhood brain and spinal tumours, and the hope is that these successes will eventually be achieved in adult patients. In the meantime, clinicians treating adult patients with brain tumour are faced with the same options, which were crudely described to me almost 50 years ago by a caring oncologist when he said *“Michael – we can lamp, we can inject, we can do both or we can do nothing!”* Sadly, for many adult patients with glioma this remains the case. Many oncologists who by virtue of the much greater prevalence of lung, breast and GI tumours will not see many patients with brain tumour, regularly get in touch with neuropathology requesting that we find a druggable target. One day soon, hopefully, the therapeutic landscape will improve as it has done in paediatric neuro-oncology. 

## Conclusions

**Figure 38: The author having received the RCSI Alumni Teaching award 2023.**
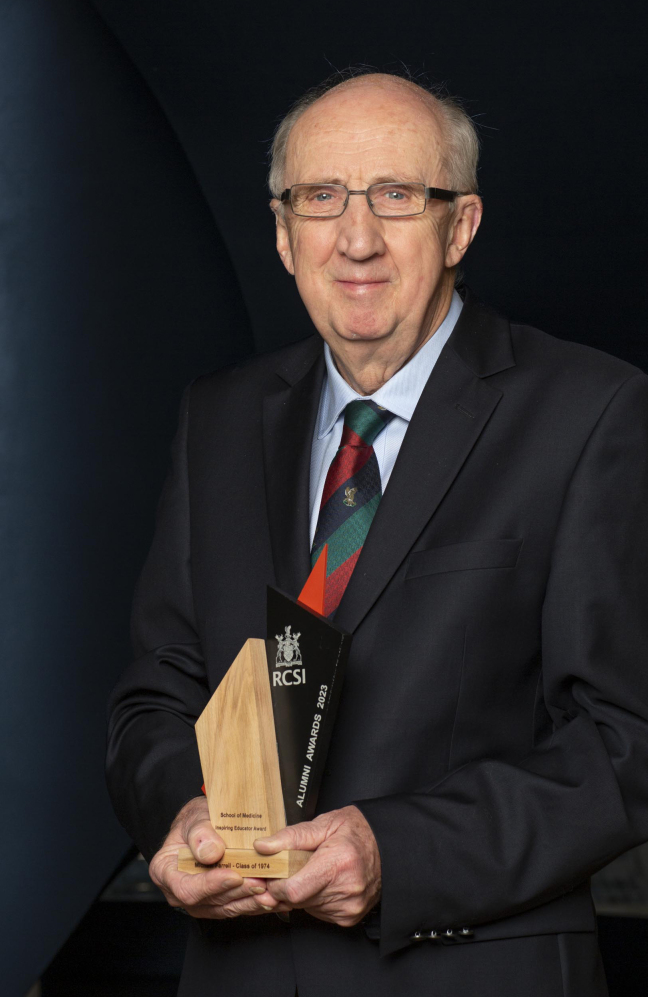


**Figure 39: The 1973 RCSI Rugby XV, Author standing left at rear.**
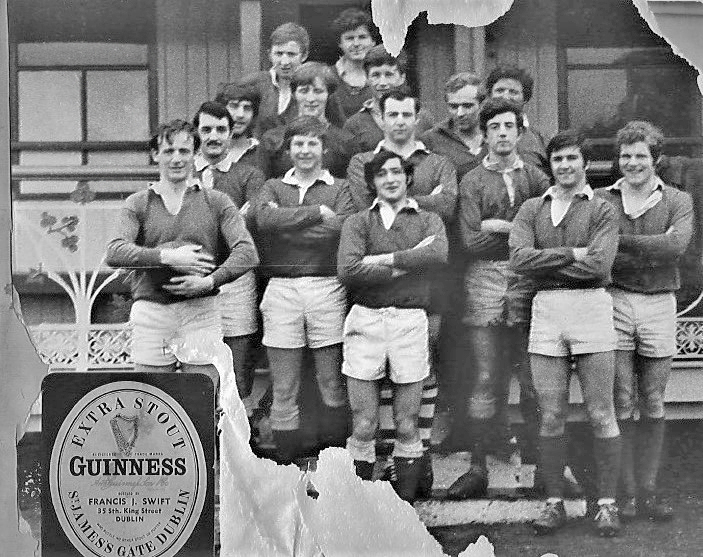


 Even though I formally retired in 2015, I remain involved in neuropathology at Beaumont and teaching at RCSI. There are regrets, not least of which is the facility with which successive Irish governments and Irish officialdom have used GDPR as an excuse not to introduce legislation that would have freed up the millions of carefully curated historic pathology samples, each with its own long term clinical outcomes, for medical research. As mentioned, the decline of autopsy pathology and especially autopsy neuropathology, in my view heralds the decline of neuropathology as a specialty, but I have not conceded in the fight to reverse this trend. On the other side, being awarded RCSI’s School of Medicine’s Inspiring Educator award in 2023 brought great satisfaction **(Fig. 38)**. Other memorable occasions include being invited to deliver the 2018 James Bull Lecture to the British Society of Neuroradiologists and the Sydney Alison Lecture at the Joint meeting of the Irish Neurological Association and The Association of British Neurologists in 2023. I was honoured to be awarded the Paula Cotter Medal by The Faculty of Pathology of the Royal College of Physicians in 2024. There were many other invitations to lecture to learned societies, all of which I was honoured to accept, whilst even though I found lecture preparation and delivery stressful, the lecture preparation work provided superb opportunities to learn and to question dogma. Collaborations with colleagues Tim Lynch **[41]**, Norman Delanty, David Henshall and Matt Campbell **[46]** have maintained my dendritic spines over the years. The friendships made through Neuropathology and the clinical neurosciences and especially with the Great Harry Vinters will remain with me to the end. At the risk of going on too long, I have not spoken of life playing **(Fig. 39**) and following rugby teams across the world or of life spent boating on the River Shannon or on the Canal Du Midi or in the various hostelries along the way. There is enough in those rugby and boating escapades for another chapter though not for publication in a scientific journal! 

Looking back now, it is terrifying to realise how quickly the years have flown. Our two children, Edward and Katie have pursued professional careers – Katie in Psychology and teaching and Edward in marine science. Sandra and I are enormously proud of their achievements. The pride in our children’s and grandchildren’s achievements would never have been realised without my wife Sandra’s constant support, as we look forward to celebrating our 50^th^ wedding anniversary in September 2026.

## Conflict of interest statement

The author declares no conflict of interest.
